# Comparing organ‐specific immune‐related adverse events for immune checkpoint inhibitors: A Bayesian network meta‐analysis

**DOI:** 10.1002/ctm2.291

**Published:** 2021-01-21

**Authors:** Meichen Li, Xue Hou, Jing Chen, Juan Yu, Meiting Chen, Na Wang, Baishen Zhang, Likun Chen

**Affiliations:** ^1^ Department of Medical Oncology Sun Yat‐Sen University Cancer Center Guangzhou P. R. China; ^2^ State Key Laboratory of Oncology in South China Guangzhou P. R. China; ^3^ Collaborative Innovation Center for Cancer Medicine Guangzhou P. R. China

Dear Editor,

Immune checkpoint inhibitor (ICI) drugs have been widely used in clinical practice and have become the first‐line treatment regimen for certain tumors, with ICI combination therapies being utilized particularly frequently.^1^, ^2^, ^3^ Although the overall incidence of organ‐specific immune‐related adverse events (irAEs) is low, such events can sometimes be severe, or even fatal and difficult to predict.^4^ However, the safety of combination regimens, which is particularly important for improving guidance of clinical treatment decisions, is not clearly understood. Here, we conducted the largest network meta‐analysis to comprehensively compare the incidence and safety rankings for common organ‐specific irAEs and general adverse events among current immunotherapeutic regimens.

We searched all randomized controlled trials involving ICI drugs published in PubMed, Web of Science, and EMBASE from 2010 to June 2020. Eligible studies must have reported detailed irAE information in patients with cancer. The study selection and data extraction procedures are described in the Supplementary Methods. Bayesian network meta‐analysis, a random‐effects, and consistency model were used to compare the risk of irAEs for different drugs. We used the odds ratio (OR) and 95% credible interval (CrI) as the effect variable for dichotomous variables. Differences with *p* values less than 0.05 or nonoverlapping 95% CrIs were considered statistically significant. Based on the OR and posterior probabilities, we ranked the risk probability of adverse events for various regimens from high to low.

A total of 61 randomized controlled trials that included 34,451 patients were enrolled in the analysis. The baseline characteristics of the included studies and the risk of bias results are shown in Tables S1 and S2. The incidence rates of any grade organ‐specific irAEs in patients who received ICI therapy were 8.25% for hypothyroidism, 3.69% for hyperthyroidism, 0.52% for hypophysitis, 1.20% for hepatitis, 2.34% for colitis, and 3.02% for pneumonitis, respectively (Table S3). As shown in Figure 1A, network meta‐analysis indicated that the risk of any grade organ‐specific irAEs was significantly higher for ICI therapy than for traditional therapy. There was no significant difference in the risk of hypothyroidism among various ICI regimens. In risk ranking, the use of two ICI drugs was associated with the highest risk of hypothyroidism. For hyperthyroidism, patients who received two ICI drugs were significantly more likely to experience hyperthyroidism of any grade than those treated with pembrolizumab, nivolumab, atezolizumab, ipilimumab, or one ICI drug combined with traditional therapy. The risk of hyperthyroidism was significantly higher with nivolumab than with ipilimumab (Figure 1A). Hypophysitis had a low incidence and mainly occurred with ipilimumab and two ICI drugs. The risk of hypophysitis was significantly higher with two ICI drugs than with pembrolizumab, nivolumab, ipilimumab, and one ICI drug combined with traditional therapy. Patients treated with ipilimumab had a significantly higher risk of hypophysitis than those treated with nivolumab. The risk of hepatitis was significantly higher with pembrolizumab, atezolizumab, two ICI drugs, and one ICI drug combined with traditional therapy than with traditional therapy. The risk of hepatitis was higher with two ICI drugs than with nivolumab. The risk of colitis was significantly higher for two ICI drugs and ipilimumab than for pembrolizumab, nivolumab, and one ICI drug combined with traditional therapy (Figure 1A). Figure 1B shows that the highest risk ranking for colitis was associated with two ICI drugs (84.4%), followed by ipilimumab (75.7%) and one ICI drug combined with traditional therapy (34.2%). There was a higher risk of pneumonitis among patients receiving pembrolizumab than those receiving durvalumab and those receiving one ICI drug combined with traditional therapy. From high to low, the risk ranking for pneumonitis was as follows: two ICI drugs, pembrolizumab, nivolumab, ipilimumab, one ICI drug combined with traditional therapy, atezolizumab, durvalumab, and traditional therapy. Detailed probabilities of organ‐specific irAEs are listed in Table S4. Pairwise meta‐analysis could be performed for 16 comparisons. Heterogeneity analysis of studies in all pairwise meta‐analysis comparisons is shown in Table S6. The pairwise meta‐analysis results are quite consistent with the network meta‐analysis results. The sensitivity analysis results are shown in Figures S3 and S4, and there was no major difference in the findings.

**FIGURE 1:**
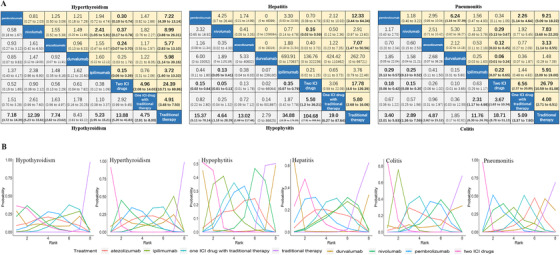
(A) Pooled odds ratios (95% credible intervals) for organic‐specific immune‐related adverse events based on Bayesian network meta‐analysis. Data in each cell are odds ratios (95% credible intervals) for the comparison of row‐defining treatment versus column‐defining treatment. Significant results are in bold. (B) Bayesian ranking curves indicate the probability of the common organic‐specific immune‐related adverse events from the highest risk to the lowest risk. ICI, immune checkpoint inhibitor

In patients who received ICI regimens, the incidence rates for general adverse events related to immune activation, such as fatigue, rash, and diarrhea, were 26.20%, 15.72%, and 21.41%, respectively (Table S6). The risk of general adverse events differed among various ICI regimens, as shown in Figure 2A. Bayesian analysis of the risk probability rankings showed that one ICI drug combined with traditional therapy was associated with the highest risk of fatigue, two ICI drugs were associated with the highest risk of rash, and ipilimumab was associated with the highest risk of diarrhea (Figure 2B).

**FIGURE 2:**
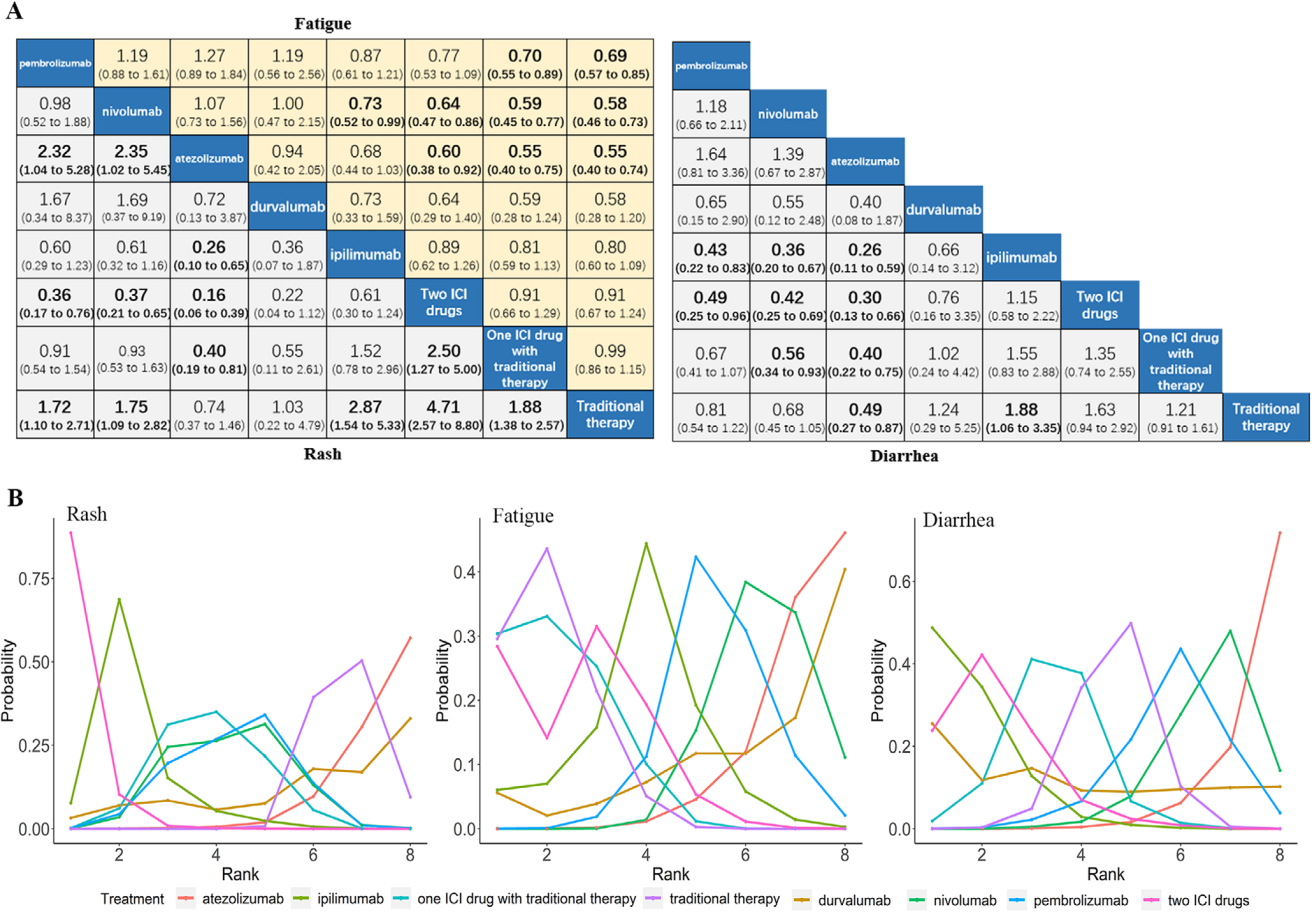
(A) Pooled odds ratios (95% credible intervals) for general adverse events related to immune activation based on Bayesian network meta‐analysis. Data in each cell are odds ratios (95% credible intervals) for the comparison of row‐defining treatment versus column‐defining treatment. Significant results are in bold. (B) Bayesian ranking curves indicate the probability of the general adverse events related to immune activation from the highest risk to the lowest risk. ICI, immune checkpoint inhibitor

The data were rearranged and merged to form the categories of ICI monotherapy, one ICI drug combined with traditional therapy, two ICI drugs, and traditional therapy. Figure 3A shows that compared with ICI monotherapy, one ICI drug combined with traditional therapy only increased the risk of fatigue and diarrhea but did not significantly increase the risk of organ‐specific irAEs. In contrast, compared with ICI monotherapy, two ICI drugs increased the risk of both organ‐specific irAEs and general adverse events (Figure 3B).

**FIGURE 3:**
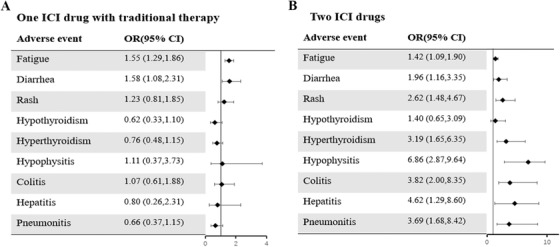
Forest plot of odds ratios (95% credible intervals) for adverse events (A) one ICI drug with traditional therapy compared to ICI monotherapy. (B) Two ICI drugs compared to ICI monotherapy. ICI, immune checkpoint inhibitor

To our knowledge, this study reports the largest network meta‐analysis to comprehensively compare the risk of organ‐specific irAEs among different ICI regimens and the largest meta‐analysis to include trials involving ICI combination regimens. Wang et al. reported that when receiving anti‐PD‐1/PD‐L1 monotherapy, 66% of patients experienced at least one adverse event, with 14% of patients experiencing at least one adverse event of grade 3 or higher.^5^ Consistent with previous studies,^6^ we revealed that the risk of organ‐specific irAEs was higher in patients who received immunotherapy than in patients who received traditional therapy and varied among different ICI regimens. For ICI monotherapy, there was no significant difference among various ICI drugs with respect to the risk of hypothyroidism, and nivolumab was associated with a higher risk of hyperthyroidism than ipilimumab. The main adverse events associated with anti‐CTLA‐4 treatment were diarrhea, colitis, and hypophysitis. The risk of hypophysitis was higher for ipilimumab than for nivolumab, and the risk of colitis was higher for ipilimumab than for pembrolizumab and nivolumab. Regarding combination therapy, the toxicity of two ICI drugs was generally higher than that of ICI monotherapy, whereas the use of one ICI drug combined with traditional therapy only increased general adverse events but did not significantly increase the risk of organ‐specific irAEs. These findings are of great significance and should be considered in drug selection and clinical decision making.

## FUNDING

This study was supported by the Guangzhou Science and Technology Program (202002020074), Sun Yat‐sen University Young Teacher Plan (19ykpy179), and Guangdong Provincial Medical Science Program (2016114134515565). The funders had no involvement in the design of the study, its implementation, or the decision to submit the article for publication.

## CONFLICTS OF INTEREST

The authors have declared no conflicts of interest.

## AUTHOR CONTRIBUTIONS

ML, XH, JC, and LC contributed to study design, literature search, data extraction, data interpretation, statistical analysis, and manuscript writing. MC, JY, NW, and BZ contributed to data extraction, data interpretation, and statistical analysis. All authors reviewed the manuscript and approved the final version submitted for publication.

## AVAILABILITY OF DATA AND MATERIALS

All data generated or analyzed during this study are included in the published article.

## Supporting information

Supporting Information
