# Supplementary material for: Comparing organ‐specific immune‐related adverse events for immune checkpoint inhibitors: A Bayesian network meta‐analysis
Source: Clin Transl Med. 2021 Jan 21;11(2):e291. doi: 10.1002/ctm2.291 (PMC7818969; doi:10.1002/ctm2.291)
Supplement: Supplementary file 1 — Supporting Information [file CTM2-11-e291-s001.docx]

**Supplementary material**

**Likun Chen et al. Comparing organ-specific immune-related adverse events for immune checkpoint inhibitors: a Bayesian network meta-analysis**

**Supplementary methods.** Search strategy, study selection and data extraction.

**Supplemental Table 1.** Characteristics of studies included for the network meta-analysis.

**Supplemental Table 2.** Risk of Bias of the randomized control trials for Bayesian network meta-analysis.

**Supplementary Table 3.** Incidence of organ specific immune-related adverse events by ICI drug.

**Supplemental Table 4.** Detailed rank and probability in the network meta-analysis.

**Supplemental Table 5.** Nodesplit analysis of the network meta-analysis.

**Supplementary Table 6.** Heterogeneity analysis in pairwise meta-analysis and network meta-analysis

**Supplementary Table 7.** Incidence of general adverse events related to immune activation by ICI drugs.

**Supplementary Table 8.** Organ-specific immune-related adverse events of one ICI drug with traditional therapy among different tumor type.

**Supplementary Figure 1.**(A) Flowchart of studies search and selection. (B) Network plot of comparisons for the Bayesian network meta-analysis. The size of the circles is proportional to the total number of patients. The width of the lines is proportional to the number of comparisons studies. ICI: immune checkpoint inhibitor.

**Supplemental Figure 2.** Pooled odds ratios (95% credible intervals) for adverse events according to one ICI drug, one ICI drug with traditional therapy, two ICI drugs and traditional therapy. Data in each cell are hazard or odds ratios (95% credible intervals) for the comparison of row-defining treatment versus column-defining treatment. Significant results are in bold. ICI: immune checkpoint inhibitor.

**Supplementary Figure 3.** Bayesian network meta-analysis based on studies excluded placebo controlled (sensitivity analysis).

**Supplementary Figure 4.** Bayesian network meta-analysis based on 45 phase III randomized control trials(sensitivity analysis).

**Supplemental References**

**Supplementary methods.**

**Search strategy and study selection**

We searched PubMed, EMBASE, and Web of Science to retrieve all the clinical trials involving ICI monotherapy or combination therapy published between 2010 and June 2020. The following keywords were used: “PD-1 inhibitor”, “PD-L1 inhibitor”，“immune checkpoint inhibitor”, “CTLA4”, “pembrolizumab”, “nivolumab”, “atezolizumab”, “durvalumab”, “ipilimumab”, and “tremelimumab”. The inclusion criteria were as follows: (1) prospective phase II/III randomized controlled trial; (2) at least one group patients received ICI therapy (ICI monotherapy, two ICI drugs or one ICI drug combined with conventional therapy); (3) detailed reports on adverse events; (4) published in English. The main exclusion criteria were as follows: (1) retrospective study, single-arm study, case report or multi-arm study in which only one ICI drug was included; (2) no adverse events reported or only overall adverse events reported, without organ specific adverse events; (3) conference abstract, news or review; (4) enrolled patients was not cancer patients, such as patients with hepatitis C infection.

**Data extraction**

Two authors (ML and XH) independently extracted and collected the following data: first author's name, year of publication, study design, blinding method, tumor type, treatment drug, criteria for adverse event evaluating, follow-up time, sample size, the number of patients with organ specific immune-related adverse events and the number of patients with general adverse events. The full text and all supplementary information was read in detail to collect comprehensive information regarding adverse events. When the same study was published multiple times, the most recent follow-up data were recorded.

| **Supplementary Table 1. Characteristics of studies included for the network meta-analysis** | | | | | | | |
| --- | --- | --- | --- | --- | --- | --- | --- |
| **First author,**  **year** | **Phase Design** | | **Cancer type** | **Arm** | **Treatment** | **Sample size (no.)** | **CTCAE version** |
| Martin Reck,2018 | III | Open-label, multicenter | NSCLC | 1  2 | Pembrolizumab  Platinum based chemotherapy | 154  150 | 4.0 |
| Jean-Louis Pujol,2019 | II | Open-label, single-center | SCLC | 1  2 | Atezolizumab  platinum–etoposide | 48  24 | 4.0 |
| Fehrenbacher, 2016 | II | Open-label, multicenter | NSCLC | 1  2 | Atezolizumab  Docetaxel | 142  135 | 4.0 |
| S.J. Antonia, 2017 | III | Double-blind, multicenter | NSCLC | 1  2 | Durvalumab  Placebo | 475  234 | 4.0 |
| D.P. Carbone,2017 | III | Double-blind, multicenter | NSCLC | 1  2 | Nivolumab  Platinum based chemotherapy | 267  263 | 4.0 |
| Yi-Long Wu, 2018 | III | Open-label, multicenter | NSCLC | 1  2 | Nivolumab  Docetaxel | 337  156 | 4.0 |
| H. Borghaei, 2015 | III | Open-label, multicenter | NSCLC | 1  2 | Nivolumab  Docetaxel | 287  268 | 4.0 |
| Julie Brahmer, 2015 | III | Open-label, multicenter | NSCLC | 1  2 | Nivolumab  Docetaxel | 131  129 | 4.0 |
| Roy S Herbst, 2016 | II/III | Open-label, multicenter | NSCLC | 1  2 | Pembrolizumab  Docetaxel | 682  309 | 4.0 |
| Tony S Mok,  2019 | III | Open-label, multicenter | NSCLC | 1  2 | Pembrolizumab  ICC | 636  615 | 4.0 |
| Achim Rittmeyer, 2016 | III | Open-label, multicenter | NSCLC | 1  2 | Atezolizumab  Docetaxel | 609  578 | 4.0 |
| Scott J Antonia, 2016 | I/II | Open-label, multicenter | SCLC | 1  2 | Nivolumab  Nivolumab + ipilimumab | 98  115 |  |
| Hellmann,  2019 | III | Open-label, multicenter | NSCLC | 1  2 | Nivolumab + ipilimumab  Platinum based chemotherapy | 576  570 | 4.0 |
| Ramaswamy Govindan, 2017 | III | Double-blind, multicenter | NSCLC | 1  2 | Ipilimumab + PTX and CBP  PTX and CBP | 388  361 | 3.0 |
| Thomas J. Lynch, 2011 | II | Double-blind, multicenter | NSCLC | 1  2 | Ipilimumab +PTX and CBP  PTX and CBP | 138  65 | 3.0 |
| Martin Reck,  2016 | III | Open-label, multicenter | SCLC | 1  2 | Ipilimumab +ETO+ DDP/CBP  ETO+ DDP/CBP | 478  476 | 3.0 |
| Corey J Langer, 2016 | II | Open-label, multicenter | NSCLC | 1  2 | Pembrolizumab  CBP and pemetrexed | 59  62 | 3.0 |
| L. Paz-Ares,  2018 | III | Double-blind, multicenter | NSCLC | 1  2 | Pembrolizumab + PTX and CBP  PTX and CBP | 278  280 | 4.0 |
| L. Gandhi,  2018 | III | Double-blind, multicenter | NSCLC | 1  2 | Pembrolizumab +pemetrexed/CBP  pemetrexed/CBP | 405  202 | 4.0 |
| Howard West,  2019 | III | Open-label, multicenter | NSCLC | 1  2 | Atezolizumab + PTX and CBP  PTX and CBP | 473  232 | 4.0 |

(Continued)

| **First author, year** | **Phase Design** | | **Cancer type** | **Arm** | **Treatment** | **Sample size** | **CTCAE version** |
| --- | --- | --- | --- | --- | --- | --- | --- |
| M**.**A. Socinski,2018 | III | Open-label, multicenter | NSCLC | 1  2 | Atezolizumab + PTX/CBP/BEV  PTX/CBP/BEV | 393  394 | 4.0 |
| Luis Paz-Ares, 2019 | III | Open-label, multicenter | SCLC | 1  2 | Durvalumab+ platinum–etoposide  platinum–etoposide | 265  266 | 4.0 |
| L. Horn,  2018 | III | Double-blind, multicenter | SCLC | 1  2 | Atezolizumab +etoposide +CBP  Placebo+ etoposide +CBP | 198  196 | 4.0 |
| J. Weber, M, 2017 | III | Double-blind, multicenter | melanoma | 1  2 | Nivolumab  Ipilimumab | 452  453 | 4.0 |
| Alexander M.M. 2018 | III | Double-blind, multicenter | melanoma | 1  2 | Pembrolizumab  Placebo | 509  502 | 4.0 |
| Caroline Robert, 2014 | III | Open-label, multicenter | melanoma | 1  2 | Nivolumab  DTIC | 206  205 | 4.0 |
| Jeffrey S Weber, 2015 | III | Open-label, multicenter | melanoma | 1  2 | Nivolumab  DTIC or PTX/CBP | 268  102 | 4.0 |
| Antoni Ribas, 2015 | II | Open-label, multicenter | melanoma | 1  2 | Pembrolizumab  ICC | 357  171 | 4.0 |
| Caroline Robert, 2015 | III | Open-label, multicenter | melanoma | 1  2 | Pembrolizumab  Ipilimumab | 555  256 | 4.0 |
| Frank Stephen Hodi,  2018 | III | Double-blind, multicenter | melanoma | 1  2  3 | Nivolumab  Nivolumab + ipilimumab  ipilimumab | 313  313  311 | 4.0 |
| Georgina V Long, 2018 | II | Open-label, multicenter | melanoma | 1  2 | Nivolumab  Nivolumab + ipilimumab | 41  35 | 4.0 |
| F Stephen Hodi, 2016 | II | Double-blind, multicenter | melanoma | 1  2 | Ipilimumab  Nivolumab + ipilimumab | 46  94 | 4.0 |
| Caroline Robert, 2014 | III | Open-label, multicenter | melanoma | 1  2 | Nivolumab  DTIC | 206  205 | 4.0 |
| Caroline Robert, 2011 | III | Open-label, multicenter | melanoma | 1  2 | Nivolumab + DTIC  DTIC | 247  251 | 3.0 |
| F. Stephen Hodi, 2014 | II | Open-label, multicenter | melanoma | 1  2 | Ipilimumab + Sargramostim  Ipilimumab | 118  120 | 4.0 |
| Georgina V Long, 2019 | III | Double-blind, multicenter | Melanoma | 1  2 | Pembrolizumab + Epacadostat  Pembrolizumab + placebo | 353  352 | 4.0 |
| Lisa Zimmer, 2020 | II | Double-blind, multicenter | Melanoma | 1  2 | Nivolumab + ipilimumab  Nivolumab | 55  56 | 4.0 |
| Ralf Gutzmer, 2020 | III | Double-blind, multicenter | Melanoma | 1  2 | Atezolizumab +vemurafenib + cobimetinib  Placebo+ vemurafenib + cobimetinib | 230  281 | 4.0 |
| Thomas Powles, 2017 | III | Open-label, multicenter | urothelial carcinoma | 1  2 | Atezolizumab  ICC | 459  443 | 4.0 |
| P. Schmid, 2018 | III | Double-blind, multicenter | Breast cancer | 1  2 | Atezolizumab + nab-paclitaxel  Placebo + nab-paclitaxel | 452  438 | 4.0 |
| (Continued) | | | | | | | |
| **First author, year** | **Phase Design** | | **Cancer type** | **Arm** | **Treatment** | **Sample size** | **CTCAE version** |
| J. Bellmunt, 2017 | III | Open-label, multicenter | urothelial carcinoma | 1  2 | Pembrolizumab  ICC | 266  255 | 4.0 |
| Eugene D Kwon, 2014 | III | Double-blind, multicenter | Prostate cancer | 1  2 | Ipilimumab  placebo | 393  396 | 4.0 |
| R.J. Motzer, 2019 | III | Open-label, multicenter | renal cell carcinoma | 1  2 | Nivolumab + ipilimumab  Sunitinib | 547  535 | 4.0 |
| B.I. Rini, 2019 | III | Open-label, multicenter | renal cell carcinoma | 1  2 | Pembrolizumab + axitinib  Sunitinib | 429  425 | 4.0 |
| Matthew D Galsky,  2020 | III | Double-blind, multicenter | urothelial carcinoma | 1  2  3 | Atezolizumab +platinum-based chemotherapy  Atezolizumab  Placebo + platinum-based chemotherapy | 453  354  390 | 4.0 |
| Brian I Rini, 2019 | III | Open-label, multicenter | renal cell carcinoma | 1  2 | Atezolizumab + bevacizumab  sunitinib | 451  446 | 4.0 |
| Yoon-Koo Kang, 2017 | III | Double-blind, multicenter | Gastric cancer | 1  2 | Nivolumab  Placebo | 330  161 | 4.0 |
| Ken Kato, 2019 | III | Open-label, multicenter | Oesophageal  cancer | 1  2 | Nivolumab  paclitaxel | 209  208 | 4.0 |
| Richard S. Finn, 2019 | III | Double-blind, multicenter | Hepatocellular  carcinoma | 1  2 | Pembrolizumab  Placebo | 279  134 | 4.0 |
| Kohei Shitara, 2018 | III | Open-label, multicenter | gastric or GEJ carcinoma | 1  2 | Pembrolizumab  PTX | 294  276 | 4.0 |
| Cathy Eng, 2019 | III | Open-label, multicenter | Colorectal cancer | 1  2  3 | Atezolizumab  Regorafenib  Atezolizumab + cobimetinib | 90  80  179 | 4.0 |
| Yung-Jue Bang, 2017 | II | Open-label, multicenter | gastric or GEJ carcinoma | 1  2 | Ipilimumab  Placebo | 57  45 | 4.0 |
| Richard S. Finn, 2020 | III | Open-label, multicenter | Hepatocellular  carcinoma | 1  2 | Atezolizumab + bevacizumab  sorafenib | 329  156 | 4.0 |
| R.L. Ferris, 2016 | III | Open-label, multicenter | head and neck cancer | 1  2 | Nivolumab  ICC | 236  111 | 4.0 |
| Ezra E W Cohen, 2018 | III | Open-label, multicenter | head and neck cancer | 1  2 | Pembrolizumab  ICC | 246  234 | 4.0 |
| Barbara Burtness, 2019 | III | Open-label, multicenter | head and neck cancer | 1  2  3 | Pembrolizumab  Pembrolizumab + platinum and 5-fluorouraci  Cetuximab + platinum and 5-fluorouraci | 300  276  287 | 4.0 |
| Antonio Omuro, | I | Open-label, multicenter | glioblastoma | 1  2 | Nivolumab  Nivolumab + ipilimumab | 10  30 | 4.0 |
| Arnaud  2019 | II | Open-label, multicenter | malignant pleural mesothelioma | 1  2 | Nivolumab  Nivolumab + ipilimumab | 63  61 | 4.0 |
| Sandra P D, 2018 | II | Open-label, multicenter | sarcoma | 1  2 | Nivolumab  Nivolumab + ipilimumab | 42  42 | 4.0 |
| (Continued) | | | | | | | |
| **First author, year** | **Phase Design** | | **Cancer type** | **Arm** | **Treatment** | **Sample size(no)** | **CTCAE version** |
| Saad Zafar Usmani, 2019 | III | Open-label, multicenter | myeloma | 1  2 | Pembrolizumab + lenalidomide and dexamethasone  lenalidomide and dexamethasone | 149  145 | 4.0 |
| P. Schmid, 2020 | III | Open-label, multicenter | Breast cancer | 1  2 | Pembrolizumab + PTX and CBP  PTX and CBP | 781  389 | 4.0 |
| Maria-Victoria Mateos, 2019 | III | Open-label, multicenter | myeloma | 1  2 | Pembrolizumab+pomalidomide dexamethasone  pomalidomide dexamethasone | 120  121 | 4.0 |

CTCAE=Common Terminology Criteria for Adverse Events; NSCLC=non small cell lung cancer; SCLC=small cell lung cancer; DTIC=dacarbazine; ICC=investigator’s choice chemotherapy; CBP=carboplatin; PTX=paclitaxel; BEV=bevacizumab; ETO=etoposide; DDP=cisplatin.

| **Supplementary Table 2. Risk of Bias of the randomized control trials for Bayesian network meta-analysis** | | | | | | | |
| --- | --- | --- | --- | --- | --- | --- | --- |
| **Study** | **Randomization** | **Allocation concealment** | **Blinding** | **Blinding of outcome assessment** | **Incomplete outcome data** | **Selective outcome reporting** | **Other sources of bias** |
| Martin Reck,2018 | Low | Low | High | High | Low | Low | Low |
| Pujol,2019 | Unclear | Unclear | High | High | Low | Low | Low |
| Fehrenbacher, 2016 | Low | Unclear | High | High | Low | unclear | Low |
| Antonia, 2017 | Low | Low | Low | Low | Unclear | Unclear | Low |
| Carbone,2017 | Low | Unclear | Low | Low | Low | Unclear | Low |
| Yi-Long Wu, 2018 | Low | Unclear | High | High | Low | High | Low |
| Borghaei, 2015 | Low | Unclear | High | Low | Low | Low | Low |
| Brahmer, 2015 | Low | Unclear | High | High | Low | Low | Low |
| Herbst, 2016 | Low | Unclear | High | Low | Low | Low | Low |
| Tony S Mok, 2019 | Low | Low | Hign | Unclear | Low | Low | Low |
| Rittmeyer, 2016 | Low | Low | High | High | Low | Low | Low |
| Antonia, 2016 | Low | Unclear | High | High | Unclear | Unclear | Low |
| Hellmann, 2019 | Low | Low | High | High | Low | Low | Low |
| Govindan, 2017 | Low | Unclear | Low | Low | Low | Low | Low |
| Lynch, 2011 | Low | Unclear | Low | Low | Unclear | Low | Low |
| Reck, 2016 | Low | Unclear | High | High | Low | Unclear | Low |
| Langer, 2016 | Low | Unclear | High | High | Unclear | Low | Low |
| Paz-Ares, 2018 | Low | Low | Low | Low | Unclear | Low | Low |
| Gandhi, 2018 | Low | Low | Low | Low | Unclear | Low | Low |
| Howardt, 2019 | Low | Unclear | High | Unclear | Unclear | Low | Low |
| Socinski,2018 | Low | Unclear | High | Unclear | Low | Low | Low |
| Paz-Ares, 2019 | Low | Low | Unclear | Unclear | Low | Low | Low |
| Horn, 2018 | Low | Low | Low | Low | Unclear | Low | Low |
| Weber, M, 2017 | Low | Unclear | Low | Low | Low | Low | Low |
| Alexander, 2018 | Low | Unclear | Low | Low | Unclear | Unclear | Low |
| Robert, 2014 | Low | Low | Low | Low | High | High | Low |
| Weber, 2015 | Low | Unclear | High | High | Unclear | Low | Low |
| Ribas, 2015 | Low | Low | High | High | Unclear | Low | Low |
| Robert, 2015(2) | Low | Low | High | High | Low | Low | Low |
| Stephen Hodi, 2018 | Low | Low | Low | Low | Unclear | Unclear | Low |
| Georgina, 2018 | Low | Unclear | High | High | Unclear | Unclear | Low |
| Hodi, 2016(2) | Low | Low | Low | Low | Unclear | Low | Low |
| Robert, 2011(3) | Low | Unclear | High | High | Unclear | Low | Low |
| Mateos, 2019 | Low | High | High | High | Low | Low | Low |
| Hodi, 2014(3) | Low | Unclear | High | High | Unclear | Low | Low |
| Georgina, 2019 | Low | Unclear | Low | Low | High | High | Low |
| Zimmer, 2020 | Low | Unclear | Low | Low | High | High | Low |
| Gutzmer, 2020 | Low | Low | Low | Low | Unclear | Unclear | Low |
| Powles, 2017 | Low | Unclear | High | High | Low | Low | Low |
| Schmid, 2018 | Low | Low | Low | Low | Unclear | Unclear | Low |
| Bellmunt, 2017 | Low | Unclear | High | High | Low | Low | Low |
| Kwon, 2014 | Low | Low | Low | Low | Unclear | Unclear | Low |
| Motzer, 2019 | Low | Low | High | High | Unclear | Unclear | Low |
| Rini, 2019 | Low | Unclear | High | High | Unclear | Low | Low |
| Galsky, 2020 | Low | Low | Low | Low | Unclear | Low | Low |
| Rini, 2019 | Low | Low | High | High | Unclear | Unclear | Low |
| Kang, 2017 | Low | Low | Low | Low | Unclear | Low | Low |
| Kato, 2019 | Low | Unclear | High | High | Unclear | Low | Low |
| Finn, 2019 | Low | Low | Low | Low | Unclear | Low | Low |
| Shitara, 2018 | Low | Unclear | High | High | Unclear | Unclear | Low |
| Eng, 2019 | Low | Low | High | High | Unclear | Unclear | Low |
| Bang, 2017 | Low | Unclear | High | High | High | High | Low |
| Richard Finn, 2020 | Low | Low | High | High | Unclear | Unclear | Low |
| Ferris, 2016 | Low | Unclear | Unclear | Unclear | Low | Low | Low |
| Cohen, 2018 | Low | Low | Unclear | Unclear | Unclear | Low | Low |
| Burtness, 2019 | Low | Low | Unclear | Unclear | Low | Low | Low |
| Omuro, 2019 | Unclear | Unclear | High | High | Low | Low | Low |
| Arnaud, 2019 | Low | Unclear | High | High | Low | Low | Low |
| Sandra P D, 2018 | Unclear | Unclear | High | High | Unclear | Low | Low |
| Usmani, 2019 | Low | Low | High | High | Unclear | Unclear | Low |
| Schmid, 2020 | Low | Unclear | High | High | Low | Low | Low |

| **Supplementary Table 3. Incidence of organ specific immune-related adverse events by ICI drug.** | | | | | | | | | | | | | |
| --- | --- | --- | --- | --- | --- | --- | --- | --- | --- | --- | --- | --- | --- |
| **Drug** | **No.** | **hypothyroidism** | | **hyperthyroidism** | | **hypophysitis** | | **colitis** | | **hepatitis** | | **pneumonitis** | |
|  |  | All (%) | Serious (%) | All (%) | Serious (%) | All (%) | Serious (%) | All (%) | Serious (%) | All (%) | Serious (%) | All (%) | Serious (%) |
| pembrolizumab | 4630 | 10.03  (8.09-12.14) | 0.04  (0.00-0.16) | 4.69  (3.49-6.05) | 0.04  (0.00-0.15) | 0.57  (0.28-0.95) | 0.35  (0.17-0.57) | 1.66  (1.14-2.26) | 0.88  (0.53-1.30) | 0.93  (0.59-1.34) | 0.66  (0.32-1.09) | 3.88  (2.65-5.33) | 1.15  (0.64-1.80) |
| Nivolumab | 3346 | 5.04  (3.40-6.94) | 0.00  (0.00-0.03) | 1.46  (0.48-2.81) | 0.00  (0.00-0.00) | 0.00  (0.00-0.10) | 0.00  (0.00-0.02) | 0.64  (0.19-1.28) | 0.09  (0.00-0.31) | 0.35  (0.05-0.83) | 0.11  (0.00-0.42) | 1.52  (0.93-2.22) | 0.12  (0.00-0.43) |
| atezolizumab | 1702 | 6.73  (3.48-10.89) | 0.03  (0.00-0.28) | 3.88  (1.65-6.88) | 0.00  (0.00-0.08) | 0.00  (0.00-0.07) | 0.00  (0.00-0.07) | 0.62  (0.14-1.34) | 0.02  (0.00-0.34) | 0.84  (0.21-1.78) | 0.07  (0.00-0.37) | 1.14  (0.23-2.56) | 0.34  (0.00-0.98) |
| durvalumab | 475 | 11.58  (8.85-14.62) | 0.21  (0.00-0.90) | 6.32  (4.29-8.69) | 0.00  (0.00-0.36) | 0.21  (0.00-0.90) | 0.00  (0.00-0.36) | 0.63  (0.08-1.60) | 0.00  (0.00-0.36) | 0.21  (0.00-0.90) | 0.00  (0.00-0.36) | 10.74  (8.10-13.69) | 4.42  (2.74-6.47) |
| ipilimumab | 1636 | 4.98  (2.83-7.64) | 0.22  (0.00-0.83) | 1.66  (0.77-2.80) | 0.06  (0.00-0.35) | 3.01  (0.77-6.40) | 1.20  (0.43-2.25) | 8.73  (7.33-10.24) | 6.34  (5.13-7.65) | 0.65  (0.11-1.50) | 0.14  (0.00-0.49) | 1.15  (0.60-1.82) | 0.32  (0.04-0.74) |
| Two ICI drugs | 1868 | 12.74  (9.43-16.44) | 0.12  (0.00-0.45) | 6.01  (2.58-10.57) | 0.09  (0.00-0.42) | 3.94  (1.26-7.77) | 1.15  (0.28-2.42) | 8.75  (3.36-16.10) | 4.70  (1.77-8.74) | 4.72  (1.94-8.45) | 2.26  (0.64-4.59) | 5.71  (4.02-7.65) | 0.95  (0.45-1.59) |
| One ICI drug with traditional therapy | 7642 | 9.26  (6.19-12.87) | 0.08  (0.01-0.19) | 4.71  (3.50-6.08) | 0.13  (0.04-0.25) | 0.35  (0.16-0.60) | 0.19  (0.07-0.35) | 2.00  (1.42-2.66) | 0.98  (0.59-1.44) | 1.76  (1.13-2.51) | 1.04  (0.69-1.46) | 3.65  (2.74-4.67) | 1.02  (0.67-1.43) |
| Total | 21299 | 8.25  (6.88-9.72) | 0.02  (0.00-0.06) | 3.69  (2.97-4.47) | 0.01  (0.00-0.04) | 0.52  (0.26-0.84) | 0.16  (0.07-0.29) | 2.34  (1.75-3.00) | 1.09  (0.72-1.52) | 1.20  (0.88-1.57) | 0.56  (0.36-0.78) | 3.02  (2.47-3.63) | 0.73  (0.52-0.96) |

ICI=immune checkpoint inhibitor

| **Supplementary Table 4.** **Detailed rank and probability in the network meta-analysis** | | | | | | | | |
| --- | --- | --- | --- | --- | --- | --- | --- | --- |
| **Treatment** | **Rank of risk*** | | | | | | | |
|  | 1 | 2 | 3 | 4 | 5 | 6 | 7 | 8 |
| **Hypothyroidism** |  |  |  |  |  |  |  |  |
| Pembrolizumab | 0.029 | 0.073 | 0.18 | 0.281 | 0.255 | 0.137 | 0.044 | 0 |
| Nivolumab | 0.219 | 0.366 | 0.243 | 0.11 | 0.044 | 0.015 | 0.003 | 0 |
| Atezolizumab | 0.093 | 0.121 | 0.183 | 0.21 | 0.172 | 0.13 | 0.091 | 0 |
| Durvalumab | 0.29 | 0.088 | 0.112 | 0.09 | 0.081 | 0.084 | 0.219 | 0.036 |
| Ipilimumab | 0.006 | 0.02 | 0.086 | 0.155 | 0.2 | 0.243 | 0.289 | 0.001 |
| Two ICI drugs | 0.362 | 0.328 | 0.178 | 0.081 | 0.035 | 0.014 | 0.003 | 0 |
| One ICI drug with traditional therapy | 0.001 | 0.003 | 0.018 | 0.074 | 0.214 | 0.377 | 0.313 | 0 |
| Traditional therapy | 0 | 0 | 0 | 0 | 0 | 0 | 0.037 | 0.963 |
| **Hyperthyroidism** |  |  |  |  |  |  |  |  |
| Pembrolizumab | 0.004 | 0.165 | 0.346 | 0.292 | 0.139 | 0.045 | 0.009 | 0 |
| Nivolumab | 0.005 | 0.449 | 0.275 | 0.143 | 0.08 | 0.044 | 0.004 | 0 |
| Atezolizumab | 0.003 | 0.087 | 0.173 | 0.223 | 0.229 | 0.184 | 0.102 | 0 |
| Durvalumab | 0.079 | 0.204 | 0.127 | 0.105 | 0.087 | 0.134 | 0.247 | 0.017 |
| Ipilimumab | 0 | 0.004 | 0.041 | 0.083 | 0.125 | 0.239 | 0.506 | 0.001 |
| Two ICI drugs | 0.909 | 0.088 | 0.003 | 0 | 0 | 0 | 0 | 0 |
| One ICI drug with traditional therapy | 0 | 0.003 | 0.035 | 0.154 | 0.34 | 0.355 | 0.114 | 0 |
| Traditional therapy | 0 | 0 | 0 | 0 | 0 | 0 | 0.018 | 0.982 |
| **Hypophysitis** |  |  |  |  |  |  |  |  |
| Pembrolizumab | 0.002 | 0.030 | 0.154 | 0.291 | 0.337 | 0.168 | 0.018 | 0.000 |
| Nivolumab | 0.000 | 0.001 | 0.011 | 0.029 | 0.110 | 0.467 | 0.377 | 0.006 |
| Atezolizumab | 0.030 | 0.092 | 0.148 | 0.159 | 0.231 | 0.245 | 0.091 | 0.004 |
| Durvalumab | 0.451 | 0.013 | 0.009 | 0.004 | 0.006 | 0.011 | 0.018 | 0.488 |
| Ipilimumab | 0.002 | 0.351 | 0.410 | 0.150 | 0.068 | 0.018 | 0.000 | 0.000 |
| Two ICI drugs | 0.510 | 0.452 | 0.031 | 0.005 | 0.001 | 0.000 | 0.000 | 0.000 |
| One ICI drug with traditional therapy | 0.005 | 0.060 | 0.237 | 0.362 | 0.247 | 0.082 | 0.008 | 0.000 |
| Traditional therapy | 0.000 | 0.000 | 0.000 | 0.000 | 0.000 | 0.009 | 0.488 | 0.502 |
| **Hepatitis** |  |  |  |  |  |  |  |  |
| Pembrolizumab | 0.09 | 0.298 | 0.314 | 0.172 | 0.081 | 0.034 | 0.012 | 0 |
| Nivolumab | 0 | 0.019 | 0.078 | 0.129 | 0.206 | 0.312 | 0.242 | 0.014 |
| Atezolizumab | 0.038 | 0.133 | 0.201 | 0.202 | 0.172 | 0.149 | 0.101 | 0.004 |
| Durvalumab | 0.627 | 0.009 | 0.006 | 0.005 | 0.004 | 0.005 | 0.011 | 0.333 |
| Ipilimumab | 0.005 | 0.033 | 0.093 | 0.151 | 0.204 | 0.276 | 0.211 | 0.025 |
| Two ICI drugs | 0.236 | 0.479 | 0.181 | 0.07 | 0.027 | 0.006 | 0.001 | 0 |
| One ICI drug with traditional therapy | 0.004 | 0.029 | 0.127 | 0.272 | 0.305 | 0.193 | 0.071 | 0 |
| Traditional therapy | 0 | 0 | 0 | 0 | 0.001 | 0.024 | 0.352 | 0.623 |
| **Colitis** |  |  |  |  |  |  |  |  |
| Pembrolizumab | 0 | 0 | 0.03 | 0.156 | 0.372 | 0.335 | 0.107 | 0 |
| Nivolumab | 0 | 0 | 0.059 | 0.096 | 0.214 | 0.393 | 0.236 | 0.001 |
| Atezolizumab | 0.021 | 0.056 | 0.294 | 0.238 | 0.171 | 0.149 | 0.068 | 0.002 |
| Durvalumab | 0.079 | 0.044 | 0.089 | 0.056 | 0.064 | 0.086 | 0.265 | 0.317 |
| Ipilimumab | 0.056 | 0.757 | 0.168 | 0.017 | 0.001 | 0 | 0 | 0 |
| Two ICI drugs | 0.844 | 0.138 | 0.017 | 0.001 | 0 | 0 | 0 | 0 |
| One ICI drug with traditional therapy | 0 | 0.004 | 0.342 | 0.434 | 0.178 | 0.036 | 0.005 | 0 |
| Traditional therapy | 0 | 0 | 0 | 0 | 0 | 0.002 | 0.319 | 0.68 |
| **Pneumonitis** |  |  |  |  |  |  |  |  |
| Pembrolizumab | 0.029 | 0.514 | 0.279 | 0.159 | 0.017 | 0.002 | 0 | 0 |
| Nivolumab | 0.001 | 0.305 | 0.394 | 0.214 | 0.063 | 0.021 | 0.002 | 0 |
| Atezolizumab | 0.001 | 0.015 | 0.044 | 0.09 | 0.194 | 0.474 | 0.172 | 0.01 |
| Durvalumab | 0.002 | 0.008 | 0.015 | 0.026 | 0.054 | 0.128 | 0.464 | 0.304 |
| Ipilimumab | 0.003 | 0.124 | 0.224 | 0.343 | 0.162 | 0.117 | 0.026 | 0 |
| Two ICI drugs | 0.965 | 0.033 | 0.002 | 0 | 0 | 0 | 0 | 0 |
| One ICI drug with traditional therapy | 0 | 0.001 | 0.041 | 0.168 | 0.51 | 0.253 | 0.027 | 0 |
| Traditional therapy | 0 | 0 | 0 | 0 | 0 | 0.005 | 0.309 | 0.686 |
| **Rash** |  |  |  |  |  |  |  |  |
| Pembrolizumab | 0.002 | 0.044 | 0.196 | 0.268 | 0.341 | 0.137 | 0.01 | 0.001 |
| Nivolumab | 0 | 0.035 | 0.245 | 0.263 | 0.314 | 0.131 | 0.011 | 0.002 |
| Atezolizumab | 0 | 0 | 0.002 | 0.006 | 0.019 | 0.097 | 0.305 | 0.571 |
| Durvalumab | 0.033 | 0.07 | 0.085 | 0.057 | 0.076 | 0.18 | 0.169 | 0.33 |
| Ipilimumab | 0.077 | 0.687 | 0.152 | 0.053 | 0.024 | 0.006 | 0.001 | 0 |
| Two ICI drugs | 0.887 | 0.103 | 0.009 | 0.001 | 0 | 0 | 0 | 0 |
| One ICI drug with traditional therapy | 0.002 | 0.061 | 0.312 | 0.35 | 0.218 | 0.056 | 0.001 | 0 |
| Traditional therapy | 0 | 0 | 0 | 0 | 0.008 | 0.394 | 0.503 | 0.095 |
| **Fatigue** |  |  |  |  |  |  |  |  |
| Pembrolizumab | 0.000 | 0.001 | 0.019 | 0.113 | 0.423 | 0.309 | 0.114 | 0.021 |
| Nivolumab | 0.000 | 0.000 | 0.000 | 0.014 | 0.153 | 0.384 | 0.337 | 0.111 |
| Atezolizumab | 0.000 | 0.000 | 0.002 | 0.012 | 0.046 | 0.120 | 0.360 | 0.461 |
| Durvalumab | 0.056 | 0.021 | 0.039 | 0.073 | 0.117 | 0.117 | 0.173 | 0.404 |
| Ipilimumab | 0.060 | 0.070 | 0.158 | 0.444 | 0.192 | 0.058 | 0.014 | 0.003 |
| Two ICI drugs | 0.284 | 0.141 | 0.315 | 0.193 | 0.053 | 0.011 | 0.001 | 0.000 |
| One ICI drug with traditional therapy | 0.304 | 0.331 | 0.253 | 0.101 | 0.012 | 0.000 | 0.000 | 0.000 |
| Traditional therapy | 0.296 | 0.436 | 0.214 | 0.051 | 0.003 | 0.000 | 0.000 | 0.000 |
| **Diarrhea** |  |  |  |  |  |  |  |  |
| Pembrolizumab | 0 | 0.003 | 0.022 | 0.067 | 0.217 | 0.436 | 0.216 | 0.038 |
| Nivolumab | 0 | 0 | 0.005 | 0.017 | 0.079 | 0.277 | 0.48 | 0.142 |
| Atezolizumab | 0 | 0 | 0.001 | 0.004 | 0.016 | 0.063 | 0.198 | 0.718 |
| Durvalumab | 0.255 | 0.118 | 0.147 | 0.093 | 0.09 | 0.096 | 0.1 | 0.102 |
| Ipilimumab | 0.488 | 0.344 | 0.128 | 0.029 | 0.009 | 0.002 | 0 | 0 |
| Two ICI drugs | 0.238 | 0.422 | 0.237 | 0.07 | 0.024 | 0.008 | 0.001 | 0 |
| One ICI drug with traditional therapy | 0.019 | 0.11 | 0.411 | 0.378 | 0.067 | 0.014 | 0.001 | 0 |
| Traditional therapy | 0 | 0.003 | 0.049 | 0.342 | 0.498 | 0.103 | 0.005 | 0 |

ICI=immune checkpoint inhibitor. * Values are presented as probability (%)

| **Supplementary Table 5.** **Nodesplit analysis of the network meta-analysis** | | | | |
| --- | --- | --- | --- | --- |
| **Nodes** | **Direct effect** | **Indirect effect** | **Network** | **P value*** |
| **hypothyroidism** |  |  |  |  |
| Pembrolizumab, ipilimumab | -1.7 (-4.0, 0.49) | -0.33 (-1.4, 0.81) | -0.33 (-1.4, 0.81) | 0.14775 |
| Pembrolizumab, one ICI drug with CT | 0.0038 (-1.5, 1.5) | -0.60 (-1.5, 0.33) | -0.41 (-1.2, 0.36) | 0.49347 |
| Pembrolizumab, CT | -1.9 (-2.7, -1.1) | -2.4 (-4.1, -0.72) | -2. (-2.7, -1.3) | 0.59454 |
| Nivolumab, ipilimumab | -0.70 (-2.3, 0.86) | -0.95 (-2.4, 0.45) | -0.86 (-1.9, 0.14) | 0.81172 |
| Nivolumab, two ICI drugs | 0.55 (-0.42, 1.5) | -1.1 (-2.8, 0.48) | 0.11 (-0.73, 0.96) | 0.07987 |
| Nivolumab, CT | -3.1 (-4.4, -2.0) | -1.5 (-3.0, -0.25) | -2.5 (-3.4, -1.7) | 0.07529 |
| Atezolizumab, one ICI drug with CT | -0.031 (-1.6, 1.5) | -1.7 (-3.6, 0.018) | -0.49 (-1.6, 0.55) | 0.15423 |
| Ipilimumab, two ICI drugs | 0.98 (-0.64, 2.6) | 1.0 (-0.50, 2.6) | 0.97 (-0.093, 2.1) | 0.97851 |
| Ipilimumab, one ICI drug with CT | -0.36 (-2.8, 2.0) | -0.021 (-1.2, 1.1) | -0.089 (-1.2, 0.95) | 0.80201 |
| Ipilimumab, CT | -2.2 (-4.7, -0.18) | -1.5 (-2.7, -0.40) | -1.6 (-2.7, -0.68) | 0.55550 |
| Two ICI drugs, CT | -1.6 (-3.3, -0.15) | -3.2 (-4.5, -2.0) | -2.6 (-3.7, -1.7) | 0.12554 |
| One ICI drug with CT, CT | -1.4 (-2.0, -0.92) | -1.9 (-3.7, -0.14) | -1.6 (-2.1, -1.1) | 0.63479 |
| **hyperthyroidism** |  |  |  |  |
| Pembrolizumab, ipilimumab | -0.81 (-2.5, 0.76) | -0.44 (-1.6, 0.91) | -0.59 (-1.5, 0.42) | 0.70240 |
| Pembrolizumab, one ICI drug with CT | 0.14 (-0.87, 1.2) | -0.73 (-1.5, 0.025) | -0.39 (-1.0, 0.20) | 0.16226 |
| Pembrolizumab, CT | -2.1 (-2.8, -1.5) | -1.9 (-3.2, -0.81) | -2.0 (-2.6, -1.5) | 0.72959 |
| Nivolumab, ipilimumab | -1.1 (-2.2, -0.0010) | -0.60 (-2.3, 0.95) | -0.94 (-1.9, -0.099) | 0.61046 |
| Nivolumab, two ICI drugs | 1.3 (0.09, 2.1) | -0.47 (-2.1, 1.0) | 0.95 (0.18, 1.7) | 0.05309 |
| Nivolumab, CT | -3.8 (-7.2, -2.1) | -1.8 (-3.0, -0.79) | -2.3 (-3.4, -1.5) | 0.06134 |
| Atezolizumab, one ICI drug with CT | 0.022 (-1.1, 1.1) | -0.76 (-2.3, 0.57) | -0.16 (-0.96, 0.62) | 0.36725 |
| Ipilimumab, two ICI drugs | 2.7 (0.99, 4.6) | 1.5 (0.34, 2.8) | 1.9 (0.95, 2.9) | 0.28358 |
| Ipilimumab, one ICI drug with CT | 0.027 (-2.2, 2.2) | 0.23 (-0.94, 1.2) | 0.20 (-0.81, 1.1) | 0.87314 |
| Ipilimumab, CT | -30.0 (-1.5e+02, -3.6) | -1.2 (-2.2, -0.29) | -1.4 (-2.4, -0.55) | 0.00566 |
| Two ICI drugs, CT | -2.4 (-3.6, -1.5) | -4.3 (-5.8, -3.1) | -3.3 (-4.4, -2.5) | 0.02371 |
| One ICI drug with CT, CT | -1.5 (-1.9, -1.1) | -1.9 (-3.3, -0.70) | -1.6 (-2.0, -1.2) | 0.47993 |
| **Hypophysitis** |  |  |  |  |
| Pembrolizumab, ipilimumab | 1.5 (-0.40, 3.7) | 0.012 (-2.1, 2.0) | 0.88 (-0.54, 2.2) | 0.24453 |
| Pembrolizumab, one ICI drug with CT | -0.53 (-2.5, 1.3) | 1.0 (-0.85, 3.0) | 0.20 (-1.1, 1.5) | 0.21889 |
| Pembrolizumab, CT | -2.8 (-5.3, -1.3) | -2.9 (-5.6, -0.96) | -2.7 (-4.3, -1.6) | 0.95140 |
| Nivolumab, ipilimumab | 2.0 (0.39, 3.8) | 1.9 (-1.2, 4.5) | 2. (0.84, 3.1) | 0.91816 |
| Nivolumab, two ICI drugs | 3.7 (2.0, 6.7) | 3.1 (0.096, 6.1) | 3.1 (2.1, 4.6) | 0.70522 |
| Nivolumab, CT | -27.0 (-62.0, -2.9) | -1.0 (-3.0, 0.79) | -1.6 (-3.5, -0.14) | 0.01013 |
| Atezolizumab, one ICI drug with CT | 1.1 (-1.5, 4.7) | -21. (-98., 0.52) | 0.36 (-1.9, 2.4) | 0.07964 |
| Ipilimumab, two ICI drugs | 0.79 (-0.56, 2.2) | 11. (1.9, 41.0) | 1.1 (0.24, 2.6) | 0.01363 |
| Ipilimumab, one ICI drug with CT | 0.077 (-2.6, 2.9) | -0.96 (-2.7, 1.1) | -0.68 (-2.1, 0.87) | 0.50197 |
| Ipilimumab, CT | -0.78 (-3.3, 1.3) | -4.5 (-6.5, -3.0) | -3.6 (-5.2, -2.3) | 0.01902 |
| Two ICI drugs, CT | -41. (-93.0, -3.8) | -4.3 (-6.4, -2.7) | -4.7 (-6.9, -3.3) | 0.07389 |
| One ICI drug with CT, CT | -3.2 (-5.4, -1.7) | -2.4 (-5.0, -0.40) | -2.9 (-4.5, -1.8) | 0.56899 |
| **Hepatitis** |  |  |  |  |
| Pembrolizumab, ipilimumab | -0.31 (-3.6, 3.0) | -1.7 (-4.2, 0.63) | -1.2 (-3.2, 0.63) | 0.48179 |
| Pembrolizumab, one ICI drug with CT | 0.37 (-2.2, 2.9) | -1.3 (-3.3, 0.45) | -0.76 (-2.3, 0.64) | 0.27213 |
| **Nodes** | **Direct effect** | **Indirect effect** | **Network** | **P value** |
| Nivolumab, ipilimumab | 0.61 (-2.4, 3.6) | 0.55 (-1.8, 2.8) | 0.26 (-1.6, 2.0) | 0.97761 |
| Nivolumab, two ICI drugs | 2.2 (0.77, 3.9) | 0.16 (-2.1, 2.7) | 1.8 (0.54, 3.3) | 0.15657 |
| Nivolumab, TT | -1.5 (-3.4, -0.049) | -0.19 (-2.7, 2.0) | -1.1 (-2.6, 0.14) | 0.32022 |
| Atezolizumab, one ICI drug with TT | -0.44 (-2.8, 1.9) | -1.0 (-4.9, 1.8) | -0.21 (-2.0, 1.5) | 0.75741 |
| Ipilimumab, two ICI drugs | 14.0 (2.4, 45.0) | 0.41 (-1.7, 2.7) | 1.5 (-0.24, 3.7) | 0.01112 |
| Ipilimumab, one ICI drug with TT | 0.0090 (-4.6, 4.7) | 0.52 (-1.4, 2.5) | 0.43 (-1.3, 2.2) | 0.83365 |
| Ipilimumab, TT | -1.7 (-5.8, 1.4) | -1.3 (-3.4, 0.66) | -1.3 (-3.1, 0.25) | 0.79553 |
| Two ICI drugs, TT | -0.94 (-3.5, 1.1) | -4.0 (-6.5, -2.2) | -2.9 (-4.9, -1.4) | 0.06872 |
| One ICI drug with TT, TT | -1.7 (-2.8, -0.85) | -3.5 (-7.0, -0.42) | -1.8 (-2.8, -0.99) | 0.27191 |
| **Colitis** |  |  |  |  |
| Pembrolizumab, ipilimumab | 1.2 (-0.069, 2.4) | 1.4 (0.40, 2.5) | 1.3 (0.58, 2.1) | 0.82398 |
| Pembrolizumab, one ICI drug with TT | 0.51 (-0.57, 1.7) | 0.35 (-0.45, 1.1) | 0.40 (-0.21, 1.0) | 0.79593 |
| Pembrolizumab, TT | -1.2 (-1.9, -0.54) | -1.4 (-2.6, -0.36) | -1.2 (-1.8, -0.70) | 0.70539 |
| Nivolumab, ipilimumab | 1.7 (0.84, 2.5) | 0.48 (-0.83, 1.7) | 1.4 (0.59, 2.0) | 0.11127 |
| Nivolumab, two ICI drugs | 1.9 (1.1, 2.8) | 1.9 (0.16, 3.4) | 1.9 (1.2, 2.5) | 0.95858 |
| Nivolumab, TT | -2.9 (-5.9, -1.1) | -0.62 (-1.6, 0.28) | -1.1 (-2.1, -0.39) | 0.02951 |
| Atezolizumab, one ICI drug with TT | -0.33 (-1.9, 1.3) | 0.14 (-2.0, 1.7) | 0.036 (-1.1, 1.1) | 0.70357 |
| Ipilimumab, two ICI drugs | 0.46 (-0.44, 1.5) | 0.65 (-0.52, 2.0) | 0.47 (-0.14, 1.3) | 0.80503 |
| Ipilimumab, one ICI drug with TT | -0.41 (-1.8, 0.93) | -1.1 (-2.1, -0.25) | -0.87 (-1.6, -0.18) | 0.36243 |
| Ipilimumab, TT | -2.5 (-4.2, -1.1) | -2.5 (-3.4, -1.8) | -2.5 (-3.2, -1.9) | 0.99669 |
| Two ICI drugs, TT | -2.6 (-4.8, -1.1) | -3.1 (-4.4, -2.3) | -3.0 (-4.0, -2.2) | 0.61054 |
| One ICI drug with TT, TT | -1.6 (-2.1, -1.1) | -2.0 (-3.2, -0.78) | -1.6 (-2.1, -1.2) | 0.52920 |
| **Pneumonitis** |  |  |  |  |
| Pembrolizumab, ipilimumab | -2.4 (-6.0, -0.054) | 0.30 (-1.1, 1.8) | -0.41 (-1.6, 0.78) | 0.05247 |
| Pembrolizumab, one ICI drug with TT | -0.65 (-1.9, 0.62) | -0.92 (-1.9, -0.045) | -0.82 (-1.5, -0.14) | 0.71321 |
| Pembrolizumab, TT | -2.1 (-2.8, -1.4) | -3.2 (-4.8, -1.8) | -2.2 (-2.9, -1.6) | 0.16742 |
| Nivolumab, ipilimumab | 0.20 (-1.2, 1.6) | -0.98 (-2.7, 0.67) | -0.31 (-1.4, 0.70) | 0.27456 |
| Nivolumab, two ICI drugs | 1.1 (0.19, 2.1) | 1.6 (-0.22, 3.6) | 1.2 (0.40, 2.0) | 0.68177 |
| Nivolumab, TT | -2.3 (-3.6, -1.2) | -1.9 (-3.6, -0.56) | -2.1 (-3.1, -1.3) | 0.70583 |
| Atezolizumab, one ICI drug with TT | -0.17 (-1.7, 1.4) | 0.47 (-1.2, 2.0) | 0.28 (-0.74, 1.3) | 0.56730 |
| Ipilimumab, two ICI drugs | 2.0 (0.44, 3.8) | 1.1 (-0.51, 2.7) | 1.5 (0.45, 2.6) | 0.43009 |
| Ipilimumab, one ICI drug with TT | -45.0 (-1.1e+02, -3.1) | -0.18 (-1.4, 1.0) | -0.41 (-1.6, 0.70) | 0.00762 |
| Ipilimumab, TT | -68.0 (-1.5e+02, -5.1) | -1.6 (-2.8, -0.49) | -1.8 (-3.0, -0.77) | 0.00726 |
| Two ICI drugs, TT | -3.2 (-5.1, -1.6) | -3.5 (-4.9, -2.3) | -3.3 (-4.4, -2.4) | 0.79067 |
| One ICI drug with TT, TT | -1.5 (-2.0, -1.0) | -0.67 (-2.5, 1.3) | -1.4 (-1.9, -1.0) | 0.39635 |
| **Rash** |  |  |  |  |
| Pembrolizumab, ipilimumab | -2.4 (-6.0, -0.054) | 0.30 (-1.1, 1.8) | -0.41 (-1.6, 0.78) | 0.05247 |
| Pembrolizumab, one ICI drug with TT | -0.65 (-1.9, 0.62) | -0.92 (-1.9, -0.045) | -0.82 (-1.5, -0.14) | 0.71321 |
| Pembrolizumab, TT | -2.1 (-2.8, -1.4) | -3.2 (-4.8, -1.8) | -2.2 (-2.9, -1.6) | 0.16742 |
| Nivolumab, ipilimumab | 0.20 (-1.2, 1.6) | -0.98 (-2.7, 0.67) | -0.31 (-1.4, 0.70) | 0.27456 |
| Nivolumab, two ICI drugs | 1.1 (0.19, 2.1) | 1.6 (-0.22, 3.6) | 1.2 (0.40, 2.0) | 0.68177 |
| Nivolumab, TT | -2.3 (-3.6, -1.2) | -1.9 (-3.6, -0.56) | -2.1 (-3.1, -1.3) | 0.70583 |
| Atezolizumab, one ICI drug with TT | -0.17 (-1.7, 1.4) | 0.47 (-1.2, 2.0) | 0.28 (-0.74, 1.3) | 0.56730 |
| **Nodes** | **Direct effect** | **Indirect effect** | **Network** | **P value** |
| Ipilimumab, one ICI drug with TT | -45.0 (-1.1e+02, -3.1) | -0.18 (-1.4, 1.0) | -0.41 (-1.6, 0.70) | 0.00762 |
| Ipilimumab, TT | -68.0 (-1.5e+02, -5.1) | -1.6 (-2.8, -0.49) | -1.8 (-3.0, -0.77) | 0.00726 |
| Two ICI drugs, TT | -3.2 (-5.1, -1.6) | -3.5 (-4.9, -2.3) | -3.3 (-4.4, -2.4) | 0.79067 |
| One ICI drug with TT, TT | -1.5 (-2.0, -1.0) | -0.67 (-2.5, 1.3) | -1.4 (-1.9, -1.0) | 0.39635 |
| **Fatigue** |  |  |  |  |
| Pembrolizumab, ipilimumab | -0.34 (-1.1, 0.40) | 0.33 (-0.059, 0.75) | 0.18 (-0.17, 0.55) | 0.10974 |
| Pembrolizumab, one ICI drug with TT | 0.16 (-0.36, 0.68) | 0.41 (0.13, 0.71) | 0.35 (0.11, 0.60) | 0.38840 |
| Pembrolizumab, TT | 0.45 (0.22, 0.69) | -0.13 (-0.69, 0.41) | 0.36 (0.15, 0.58) | 0.05057 |
| Nivolumab, ipilimumab | -0.21 (-0.67, 0.25) | 0.69 (0.27, 1.1) | 0.29 (-0.029, 0.63) | 0.00511 |
| Nivolumab, two ICI drugs | 0.65 (0.29, 1.0) | -0.056 (-0.52, 0.41) | 0.42 (0.13, 0.73) | 0.01877 |
| Nivolumab, TT | 0.45 (0.19, 0.72) | 0.52 (0.070, 0.99) | 0.47 (0.24, 0.70) | 0.79865 |
| Atezolizumab, one ICI drug with TT | 0.39 (-0.17, 0.96) | 0.72 (0.29, 1.2) | 0.60 (0.27, 0.94) | 0.36378 |
| Ipilimumab, two ICI drugs | 0.12 (-0.46, 0.67) | 0.12 (-0.36, 0.61) | 0.13 (-0.24, 0.50) | 0.99663 |
| Ipilimumab, one ICI drug with TT | -0.046 (-0.87, 0.77) | 0.22 (-0.16, 0.58) | 0.17 (-0.17, 0.50) | 0.56237 |
| Ipilimumab, TT | -0.53 (-1.1, 0.0083) | 0.47 (0.12, 0.79) | 0.18 (-0.14, 0.48) | 0.00313 |
| Two ICI drugs, TT | 0.42 (-0.044, 0.88) | -0.21 (-0.62, 0.18) | 0.051 (-0.27, 0.35) | 0.06136 |
| One ICI drug with CT, TT | -0.025 (-0.19, 0.14) | 0.35 (-0.25, 0.94) | 0.0080 (-0.14, 0.16) | 0.22240 |
| **Diarrhea** |  |  |  |  |
| Pembrolizumab, ipilimumab | 0.45 (-0.91, 1.8) | 0.98 (0.24, 1.7) | 0.86 (0.21, 1.5) | 0.50395 |
| Pembrolizumab, one ICI drug with TT | 0.38 (-0.60, 1.4) | 0.39 (-0.15, 0.92) | 0.41 (-0.061, 0.87) | 0.99445 |
| Pembrolizumab, TT | 0.31 (-0.14, 0.76) | -0.32 (-1.4, 0.72) | 0.21 (-0.19, 0.62) | 0.26826 |
| Nivolumab, ipilimumab | 0.79 (-0.18, 1.8) | 1.2 (0.41, 2.0) | 0.99 (0.40, 1.6) | 0.52024 |
| Nivolumab, two ICI drugs | 1.0 (0.40, 1.6) | 0.48 (-0.44, 1.4) | 0.86 (0.36, 1.4) | 0.34700 |
| Nivolumab, TT | 0.31 (-0.16, 0.78) | 0.47 (-0.37, 1.3) | 0.35 (-0.060, 0.76) | 0.73604 |
| Atezolizumab, one ICI drug with TT | 1.0 (0.028, 2.1) | 0.94 (0.11, 1.8) | 0.90 (0.29, 1.5) | 0.87950 |
| Ipilimumab, two ICI drugs | 0.46 (-0.53, 1.5) | -0.58 (-1.4, 0.29) | -0.13 (-0.79, 0.53) | 0.11968 |
| Ipilimumab, one ICI drug with TT | -0.15 (-1.6, 1.3) | -0.52 (-1.2, 0.15) | -0.45 (-1.1, 0.16) | 0.63685 |
| Ipilimumab, TT | -1.9 (-3.0, -0.80) | -0.20 (-0.84, 0.44) | -0.64 (-1.2, -0.081) | 0.00898 |
| Two ICI drugs, TT | 0.21 (-0.72, 1.1) | -0.88 (-1.6, -0.22) | -0.51 (-1.1, 0.029) | 0.05804 |
| One ICI drug with TT, TT | -0.18 (-0.48, 0.11) | -0.10 (-1.2, 0.95) | -0.19 (-0.47, 0.090) | 0.88514 |

ICI= immune checkpoint inhibitor; TT=traditional therapy.

* P < 0·05 indicates a significant inconsistency between the direct effect and indirect effects.

| **Supplementary Table 6. Heterogeneity analysis in pairwise meta-analysis and network meta-analysis** | | | |
| --- | --- | --- | --- |
|  | **I^2^ (Pair-wise)** | **I^2^ (Network)** | **P value** |
| **Hypothyroidism** |  |  |  |
| Pembrolizumab, ipilimumab | - | 88.3 | 0.136 |
| Pembrolizumab, One ICI drug with TT | 5.0 | 70.7 | 0.488 |
| Pembrolizumab, TT | 64.5 | 64.6 | 0.363 |
| Nivolumab, Ipilimumab | 0.0 | 14.1 | 0.725 |
| Nivolumab, Two ICI drugs | 12.0 | 54.6 | 0.05 |
| Nivolumab, TT | 22.0 | 14.3 | 0.122 |
| Atezolizumab, One ICI drug with TT | 0.0 | 62.4 | 0.366 |
| Atezolizumab, TT | 62.8 | 62.0 | 0.420 |
| Durvalumab, TT | - | - | - |
| Ipilimumab, Two ICI drugs | 69.3 | 37.9 | 0.982 |
| Ipilimumab, One ICI drug with TT | - | 0.0 | 0.782 |
| Ipilimumab, TT | 0.0 | 0.0 | 0.552 |
| Two ICI drugs, TT | 99.5 | 99.6 | 0.08 |
| One ICI drug with TT, TT | 89.8 | 91.3 | 0.302 |
| Total | 88.5 | 92.0 |  |
| **Hyperthyroidism** |  |  |  |
| Pembrolizumab, ipilimumab | - | 0.0 | 0.720 |
| Pembrolizumab, One ICI drug with TT | 23.5 | 60.4 | 0.112 |
| Pembrolizumab, TT | 0.0 | 0.0 | 0.081 |
| Nivolumab, Ipilimumab | 0.0 | 0.0 | 0.995 |
| Nivolumab, Two ICI drugs | 0.0 | 0.0 | 0.722 |
| Nivolumab, TT | 38.5 | 21.2 | 0.207 |
| Atezolizumab, One ICI drug with TT | 43.7 | 50.7 | 0.235 |
| Atezolizumab, TT | 4.5 | 3.6 | 0.231 |
| Durvalumab, TT | - | - | - |
| Ipilimumab, Two ICI drugs | 0.0 | 0.0 | 0.265 |
| Ipilimumab, One ICI drug with TT | - | 0.0 | 0.799 |
| Ipilimumab, TT | 0.0 | 2.7 | 0.04 |
| Two ICI drugs, TT | 85.1 | 99.3 | 0.07 |
| One ICI drug with TT, TT | 17.5 | 33.4 | 0.512 |
| Total | 13.8 | 35.7 |  |
| **Hypophysitis** |  |  |  |
| Pembrolizumab, ipilimumab | - | 46.4 | 0.255 |
| Pembrolizumab, One ICI drug with TT | 0.0 | 0.8 | 0.222 |
| Pembrolizumab, TT | 10.8 | 1.7 | 0.840 |
| Nivolumab, Ipilimumab | 0.0 | 0.0 | 0.848 |
| Nivolumab, Two ICI drugs | 31.7 | 23.4 | 0.301 |
| Nivolumab, TT | 0.0 | 0.0 | 0.300 |
| Atezolizumab, One ICI drug with TT | 0.0 | 0.0 | 0.341 |
| Atezolizumab, TT | 0.0 | 0.0 | 0.452 |
| Durvalumab, TT | - | - | - |
| Ipilimumab, Two ICI drugs | 0.0 | 0.0 | 0.236 |
| Ipilimumab, One ICI drug with TT | - | 0.0 | 0.580 |
| Ipilimumab, TT | 0.0 | 84.0 | 0.001 |
| Two ICI drugs, TT | 0.0 | 38.6 | 0.183 |
| One ICI drug with TT, TT | 0.0 | 0.0 | 0.872 |
| Total | 0.0 | 0.0 |  |
| **Hepatitis** |  |  |  |
| Pembrolizumab, ipilimumab | - | 85.1 | 0.064 |
| Pembrolizumab, One ICI drug with TT | 15.5 | 50.2 | 0.261 |
| Pembrolizumab, TT | 76.0 | 75.5 | 0.554 |
| Nivolumab, Ipilimumab | 24.9 | 92.7 | 0.006 |
| Nivolumab, Two ICI drugs | 53.7 | 58.5 | 0.016 |
| Nivolumab, TT | 67.0 | 63.4 | 0.624 |
| Atezolizumab, One ICI drug with TT | 0.0 | 62.4 | 0.328 |
| Atezolizumab, TT | 46.3 | 44.3 | 0.316 |
| Durvalumab, TT | - | - | - |
| Ipilimumab, Two ICI drugs | 79.4 | 59.4 | 0.949 |
| Ipilimumab, One ICI drug with TT | - | 5.91 | 0.413 |
| Ipilimumab, TT | 75.8 | 91.8 | 0.003 |
| Two ICI drugs, TT | 0.0 | 89.0 | 0.005 |
| One ICI drug with TT, TT | 11.2 | 13.4 | 0.279 |
| Total | 54.2 | 64.2 |  |
| **Colitis** |  |  |  |
| Pembrolizumab, ipilimumab | - | 0.0 | 0.913 |
| Pembrolizumab, One ICI drug with TT | 0.0 | 0.0 | 0.832 |
| Pembrolizumab, TT | 18.4 | 9.2 | 0.975 |
| Nivolumab, Ipilimumab | 0.0 | 0.0 | 0.466 |
| Nivolumab, Two ICI drugs | 0.0 | 0.0 | 0.689 |
| Nivolumab, TT | 47.8 | 44.7 | 0.198 |
| Atezolizumab, One ICI drug with TT | 0.0 | 0.0 | 0.694 |
| Atezolizumab, TT | 0.0 | 0.0 | 0.374 |
| Durvalumab, TT | - | - | - |
| Ipilimumab, Two ICI drugs | 65.1 | 26.5 | 0.940 |
| Ipilimumab, One ICI drug with TT | - | 0.0 | 0.503 |
| Ipilimumab, TT | 39.1 | 0.0 | 0.919 |
| Two ICI drugs, TT | 41.1 | 0.0 | 0.716 |
| One ICI drug with TT, TT | 17.9 | 14.9 | 0.867 |
| Total | 18.4 | 12.0 |  |
| **Pneumonitis** |  |  |  |
| Pembrolizumab, ipilimumab | - | 65.7 | 0.164 |
| Pembrolizumab, One ICI drug with TT | 67.3 | 45.3 | 0.755 |
| Pembrolizumab, TT | 35.8 | 35.9 | 0.488 |
| Nivolumab, Ipilimumab | 0.0 | 0.0 | 0.412 |
| Nivolumab, Two ICI drugs | 17.0 | 6.1 | 0.756 |
| Nivolumab, TT | 26.0 | 14.4 | 0.752 |
| Atezolizumab, One ICI drug with TT | 0.0 | 0.0 | 0.481 |
| Atezolizumab, TT | 0.0 | 0.0 | 0.631 |
| Durvalumab, TT | - | - | - |
| Ipilimumab, Two ICI drugs | 74.5 | 45.1 | 0.459 |
| Ipilimumab, One ICI drug with TT | - | 43.2 | 0.132 |
| Ipilimumab, TT | 0.0 | 0.0 | 0.093 |
| Two ICI drugs, TT | 74.8 | 55.8 | 0.864 |
| One ICI drug with TT, TT | 35.7 | 29.7 | 0.588 |
| Total | 31.4 | 30.0 |  |
| **Rash** |  |  |  |
| Pembrolizumab, ipilimumab | - | 80.3 | 0.498 |
| Pembrolizumab, One ICI drug with TT | 0.0 | 22.6 | 0.696 |
| Pembrolizumab, TT | 91.1 | 91.0 | 0.234 |
| Nivolumab, Ipilimumab | 80.4 | 65.5 | 0.544 |
| Nivolumab, Two ICI drugs | 76.7 | 67.6 | 0.343 |
| Nivolumab, TT | 74.2 | 70.7 | 0.497 |
| Atezolizumab, One ICI drug with TT | 85.2 | 78.7 | 0.150 |
| Atezolizumab, TT | 60.0 | 71.8 | 0.358 |
| Durvalumab, TT | - | - | - |
| Ipilimumab, Two ICI drugs | 0.0 | 0.0 | 0.920 |
| Ipilimumab, One ICI drug with TT | - | 18.3 | 0.658 |
| Ipilimumab, TT | 0.0 | 0.0 | 0.538 |
| Two ICI drugs, TT | 82.4 | 93.2 | 0.219 |
| One ICI drug with TT, TT | 87.2 | 86.8 | 0.647 |
| Total | 84.4 | 84.5 |  |
| **Fatigue** |  |  |  |
| Pembrolizumab, ipilimumab | - | 85.1 | 0.064 |
| Pembrolizumab, One ICI drug with TT | 15.5 | 50.2 | 0.261 |
| Pembrolizumab, TT | 76.0 | 75.3 | 0.725 |
| Nivolumab, Ipilimumab | 24.9 | 92.7 | 0.006 |
| Nivolumab, Two ICI drugs | 53.7 | 58.5 | 0.163 |
| Nivolumab, TT | 67.0 | 63.4 | 0.624 |
| Atezolizumab, One ICI drug with TT | 0.0 | 0.0 | 0.328 |
| Atezolizumab, TT | 46.3 | 44.3 | 0.291 |
| Durvalumab, TT | - | - | - |
| Ipilimumab, Two ICI drugs | 79.4 | 59.3 | 0.949 |
| Ipilimumab, One ICI drug with TT | - | 5.9 | 0.413 |
| Ipilimumab, TT | 75.8 | 91.8 | 0.037 |
| Two ICI drugs, TT | 0.0 | 89.0 | 0.005 |
| One ICI drug with TT, TT | 11.2 | 13.4 | 0.679 |
| Total | 54.2 | 64.2 |  |
| **Diarrhea** |  |  |  |
| Pembrolizumab, ipilimumab | - | 76.4 | 0.517 |
| Pembrolizumab, One ICI drug with TT | 88.0 | 76.7 | 0.937 |
| Pembrolizumab, TT | 82.1 | 80.8 | 0.309 |
| Nivolumab, Ipilimumab | 56.3 | 43.5 | 0.550 |
| Nivolumab, Two ICI drugs | 65.5 | 58.4 | 0.473 |
| Nivolumab, TT | 76.9 | 73.9 | 0.780 |
| Atezolizumab, One ICI drug with TT | 95.6 | 90.7 | 0.778 |
| Atezolizumab, TT | 57.0 | 63.9 | 0.165 |
| Durvalumab, TT | - | - | - |
| Ipilimumab, Two ICI drugs | 0.0 | 85.2 | 0.129 |
| Ipilimumab, One ICI drug with TT | - | 18.0 | 0.644 |
| Ipilimumab, TT | 88.4 | 92.2 | 0.121 |
| Two ICI drugs, TT | 98.5 | 98.6 | 0.068 |
| One ICI drug with TT, TT | 91.3 | 90.9 | 0.847 |
| Total | 88.1 | 89.2 |  |

| **Supplementary Table 7. Incidence of general adverse events related to immune activation by ICI drugs.** | | | | | | | | |
| --- | --- | --- | --- | --- | --- | --- | --- | --- |
| **Drug** | **No.** | **Rash** | | **Fatigue** | | **Diarrhea** | | |
|  |  | All (%) | Serious (%) | All (%) | Serious (%) | All (%) | | Serious (%) |
| pembrolizumab | 4630 | 10.62  (8.09-12.14) | 0.36  (0.14-0.66) | 19.08  (14.22-24.46) | 1.32  (0.74-2.04) | 11.71  (8.79-14.97) | 0.95  (0.59-1.39) | |
| Nivolumab | 3346 | 10.14  (3.40-6.94) | 0.08  (0.00-0.29) | 21.00  (15.20-27.43) | 0.41  (0.16-0.74) | 13.68  (9.01-19.10) | 0.61  (0.15-1.27) | |
| atezolizumab | 1702 | 6.86  (3.48-10.89) | 0.03  (0.00-0.29) | 18.96  (13.24-25.41) | 1.56  (0.45-3.19) | 11.56  (8.60-14.89) | 0.31  (0.00-1.10) | |
| durvalumab | 475 | 7.79  (5.54-10.38) | 0.21  (0.00-0.90) | 13.05  (10.16-16.24) | 0.21  (0.00-0.90) | 9.68  (7.18-12.52) | 0.42  (0.01-1.26) | |
| ipilimumab | 1636 | 24.33  (19.19-29.86) | 2.04  (0.61-4.12) | 31.38  (24.17-39.06) | 2.49  (0.54-5.55) | 35.72  (29.04-42.68) | 7.33  (3.81-11.80) | |
| Two ICI drugs | 1868 | 26.35  (19.96-33.26) | 1.62  (0.75-2.72) | 42.01  (30.94-53.50) | 2.75  (1.28-4.66) | 37.31  (27.01-48.21) | 5.74  (2.88-9.39) | |
| One ICI drug with traditional therapy | 7642 | 19.84  (16.21-23.72) | 1.35  (0.76-2.08) | 29.35  (24.77-34.15) | 3.25  (2.40-4.21) | 26.84  (21.99-31.97) | 3.57  (2.54-4.75) | |
| Total | 21299 | 15.72  (6.88-9.72) | 0.71  (0.46-1.00) | 26.20  (23.43-29.06) | 1.90  (1.45-2.39) | 21.41  (18.41-24.57) | 2.39  (1.75-3.12) | |

| **Supplementary Table 8. Organ-specific immune-related adverse events of one ICI drug with traditional therapy among different tumor type.** | | | | | | | |
| --- | --- | --- | --- | --- | --- | --- | --- |
|  | **No.** | **Hypothyroidism**  **(%)** | **Hyperthyroidism**  **(%)** | **Hypophysitis**  **(%)** | **Hepatitis**  **(%)** | **Colitis**  **(%)** | **Pneumonitis**  **(%)** |
| Lung cancer | 3075 | **6.46**  **(2.85-11.32)** | **3.69**  **(2.36-5.28)** | 0.50  (0.24-0.82) | 1.67  (0.94-2.58) | 2.27  (1.30-3.47) | **3.80**  **(2.64-5.15)** |
| Melanoma | 948 | 7.39  (2.07-15.46) | 5.12  (0.52-13.56) | 0.24  (0.00-1.02) | 1.33  (0.65-2.22) | 3.09  (1.06-6.03) | 4.25  (0.57-10.73) |
| Urinary system cancer | 1333 | 21.86  (9.52-37.49) | **8.73**  **(5.45-12.69)** | 0.45  (0.00-1.49) | 1.83  (1.07-2.77) | 1.50  (0.23-3.70) | 2.70  (1.88-3.66) |
| Gastrointestinal cancer | 508 | 9.70  (6.97-12.81) | 4.31  (2.68-6.29) | 0.00  (0.00-0.36) | 5.73  (0.00-22.42) | 1.94  (0.87-3.39) | **1.16**  **(0.35-2.35)** |
| Head and neck cancer | 276 | **15.94**  **(11.84-20.51)** | 4.35  (2.22-7.11) | 0.36  (0.00-1.55) | 0.36  (0.00-1.55) | 2.54  (0.96-4.78) | **5.43**  **(3.03-8.46)** |
| other | 1502 | 9.45  (4.47-15.97) | 4.41  (3.40-5.53) | 0.23  (0.00-1.54) | 1.41  (0.83-2.11) | 1.15  (0.59-1.85) | 4.55  (2.20-7.63) |

**Supplementary Figure 1. (A)** Flowchart of studies search and selection. **(B)** Network plot of comparisons for the Bayesian network meta-analysis. The size of the circles is proportional to the total number of patients. The width of the lines is proportional to the number of comparisons studies. ICI: immune checkpoint inhibitor.


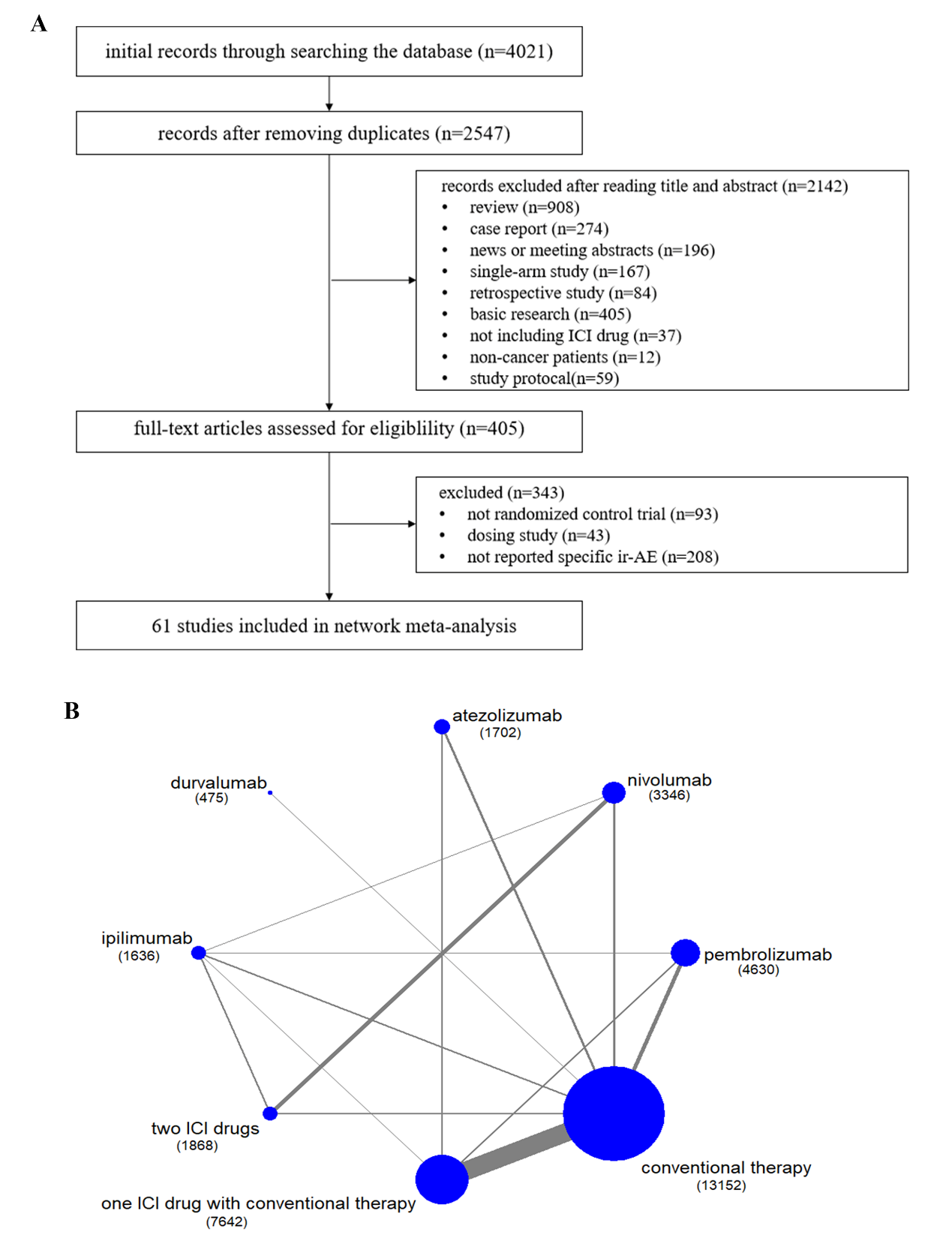


**Supplementary Figure 2.** Pooled odds ratios (95% credible intervals) for adverse events according to one ICI drug, one ICI drug with traditional therapy, two ICI drugs and traditional therapy. Data in each cell are hazard or odds ratios (95% credible intervals) for the comparison of row-defining treatment versus column-defining treatment. Significant results are in bold. ICI: immune checkpoint inhibitor.


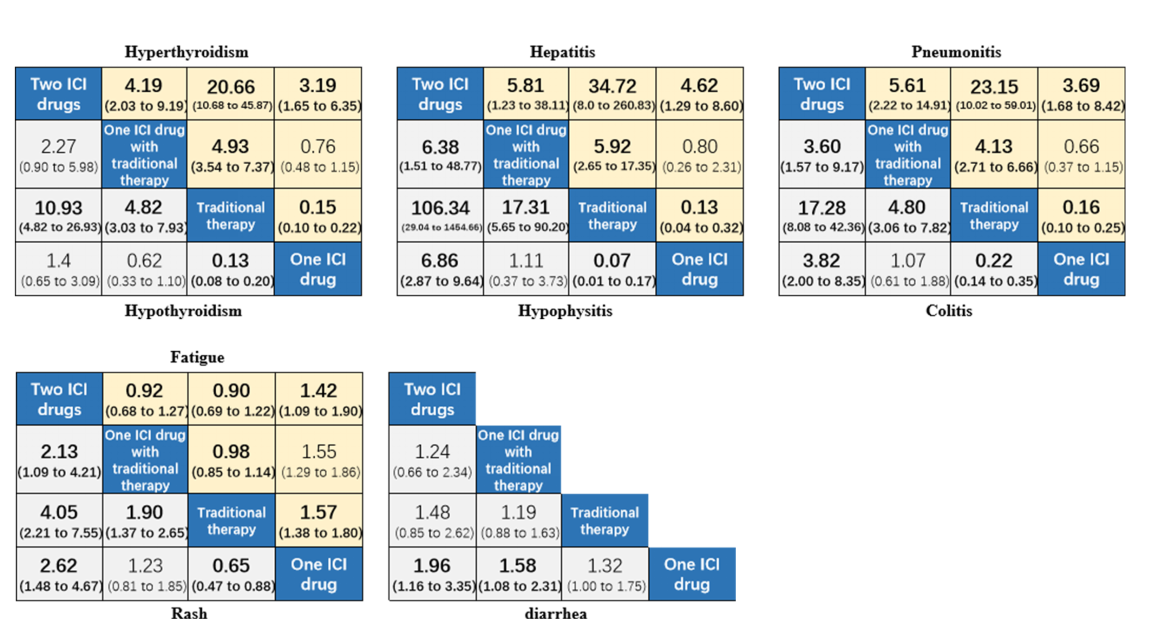


**Supplementary Figure 3.** Bayesian network meta-analysis based on studies excluded placebo controlled (sensitivity analysis).


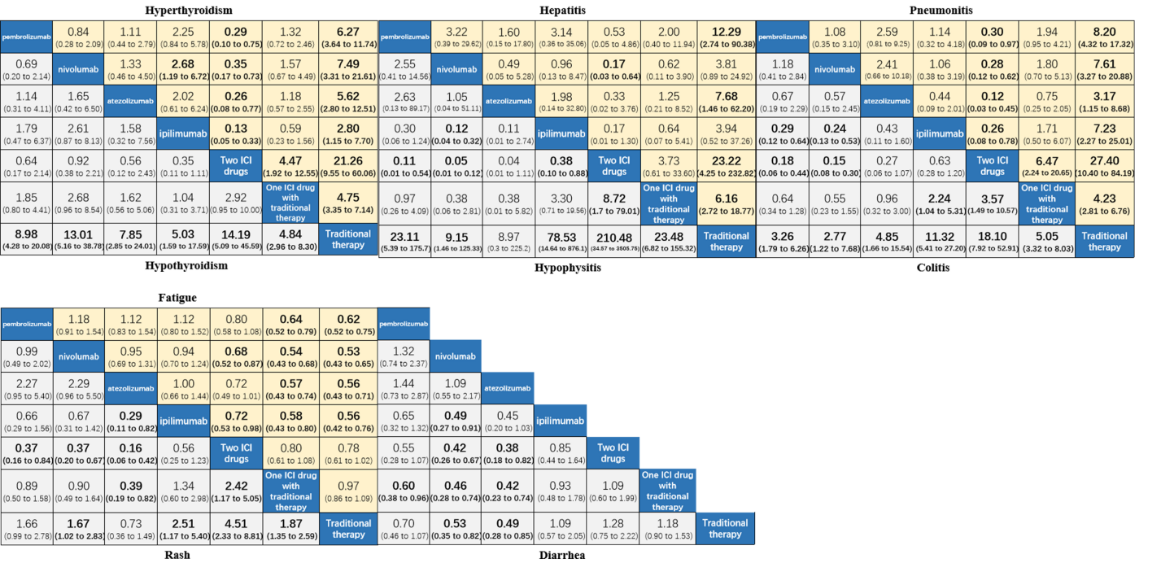


**Supplementary Figure 4.** Bayesian network meta-analysis based on 45 phase III randomized control trials (sensitivity analysis).


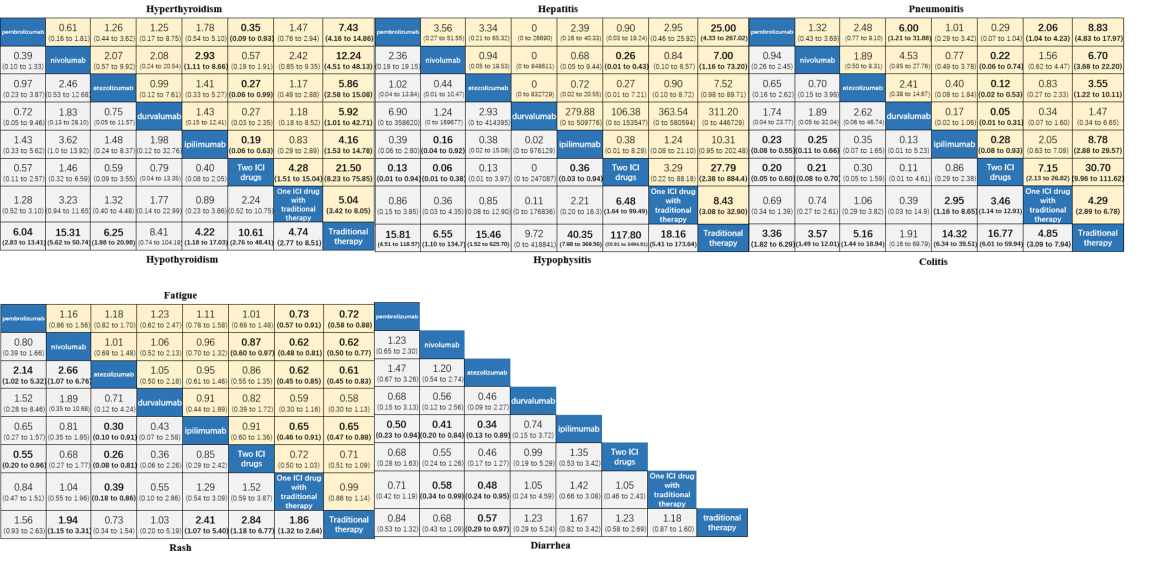


**Supplementary References**

1. Pujol J L, Greillier L, Audigier-Valette C, et al. A randomized non-comparative phase II study of anti-programmed cell death-ligand 1 atezolizumab or chemotherapy as second-line therapy in patients with small cell lung cancer: results from the IFCT-1603 trial. J Thorac Oncol. 2019; 14(5): 903-913.
2. Weber J, Mandala M, Del Vecchio M, et al. Adjuvant nivolumab versus ipilimumab in resected stage III or IV melanoma. N Engl J Med. 2017; 377(19): 1824-1835.
3. Eggermont A M M, Blank C U, Mandala M, et al. Adjuvant pembrolizumab versus placebo in resected stage III melanoma. N Engl J Med. 2018; 378(19): 1789-1801.
4. Eng C, Kim T W, Bendell J, et al. Atezolizumab with or without cobimetinib versus regorafenib in previously treated metastatic colorectal cancer (IMblaze370): a multicentre, open-label, phase 3, randomised, controlled trial. Lancet Oncol. 2019; 20(6): 849-861.
5. Antonia S J, Villegas A, Daniel D, et al. Durvalumab after chemoradiotherapy in stage III non–small-cell lung cancer. N Engl J Med. 2017; 377(20): 1919-1929.
6. Antonia S J, Villegas A, Daniel D, et al. Overall survival with durvalumab after chemoradiotherapy in stage III NSCLC. N Engl J Med. 2018; 379(24): 2342-2350.
7. Carbone D P, Reck M, Paz-Ares L, et al. First-line nivolumab in stage IV or recurrent non–small-cell lung cancer. N Engl J Med. 2017; 376(25): 2415-2426.
8. Ferris R L, Blumenschein Jr G, Fayette J, et al. Nivolumab for recurrent squamous-cell carcinoma of the head and neck. N Engl J Med, 2016; 375: 1856-1867.
9. Kang Y K, Boku N, Satoh T, et al. Nivolumab in patients with advanced gastric or gastro-oesophageal junction cancer refractory to, or intolerant of, at least two previous chemotherapy regimens (ONO-4538-12, ATTRACTION-2): a randomised, double-blind, placebo-controlled, phase 3 trial. Lancet. 2017; 390(10111): 2461-2471.
10. Robert C, Long G V, Brady B, et al. Nivolumab in previously untreated melanoma without BRAF mutation. N Engl J Med. 2015; 372(4): 320-330.
11. Borghaei H, Paz-Ares L, Horn L, et al. Nivolumab versus docetaxel in advanced nonsquamous non–small-cell lung cancer. N Engl J Med. 2015; 373(17): 1627-1639.
12. Brahmer J, Reckamp K L, Baas P, et al. Nivolumab versus docetaxel in advanced squamous-cell non–small-cell lung cancer. N Engl J Med. 2015; 373(2): 123-135.
13. Harrington K J, Ferris R L, Blumenschein Jr G, et al. Nivolumab versus standard, single-agent therapy of investigator's choice in recurrent or metastatic squamous cell carcinoma of the head and neck (CheckMate 141): health-related quality-of-life results from a randomised, phase 3 trial. Lancet Oncol. 2017; 18(8): 1104-1115.
14. Omuro A, Vlahovic G, Lim M, et al. Nivolumab with or without ipilimumab in patients with recurrent glioblastoma: results from exploratory phase I cohorts of CheckMate 143. Neuro Oncol. 2018; 20(5): 674-686.
15. D'Angelo S P, Mahoney M R, Van Tine B A, et al. Nivolumab with or without ipilimumab treatment for metastatic sarcoma (Alliance A091401): two open-label, non-comparative, randomised, phase 2 trials. Lancet Oncol. 2018; 19(3): 416-426.
16. Bellmunt J, De Wit R, Vaughn D J, et al. Pembrolizumab as second-line therapy for advanced urothelial carcinoma. N Engl J Med. 2017; 376(11): 1015-1026.
17. Finn R S, Ryoo B Y, Merle P, et al. Pembrolizumab as second-line therapy in patients with advanced hepatocellular carcinoma in KEYNOTE-240: a randomized, double-blind, phase III trial. J Clin Oncol. 2020; 38(3): 193-202.
18. Reck M, Rodríguez-Abreu D, Robinson A G, et al. Pembrolizumab versus chemotherapy for PD-L1–positive non–small-cell lung cancer. N Engl J Med. 2016; 375: 1823-1833.
19. Mok T S K, Wu Y L, Kudaba I, et al. Pembrolizumab versus chemotherapy for previously untreated, PD-L1-expressing, locally advanced or metastatic non-small-cell lung cancer (KEYNOTE-042): a randomised, open-label, controlled, phase 3 trial. Lancet. 2019; 393(10183): 1819-1830.
20. Herbst R S, Baas P, Kim D W, et al. Pembrolizumab versus docetaxel for previously treated, PD-L1-positive, advanced non-small-cell lung cancer (KEYNOTE-010): a randomised controlled trial. Lancet. 2016; 387(10027): 1540-1550.
21. Robert C, Schachter J, Long G V, et al. Pembrolizumab versus ipilimumab in advanced melanoma. N Engl J Med. 2015; 372(26): 2521-2532.
22. Cohen E E W, Soulières D, Le Tourneau C, et al. Pembrolizumab versus methotrexate, docetaxel, or cetuximab for recurrent or metastatic head-and-neck squamous cell carcinoma (KEYNOTE-040): a randomised, open-label, phase 3 study. Lancet. 2019; 393(10167): 156-167.
23. Reck M, Rodríguez-Abreu D, Robinson A, et al. Updated analysis of KEYNOTE-024: pembrolizumab versus platinum-based chemotherapy for advanced non-small-cell lung cancer with PD-L1 tumor proportion score of 50% or greater. J Clin Oncol. 2019; 37(7):537-546
24. Wu Y L, Lu S, Cheng Y, et al. Nivolumab versus docetaxel in a predominantly Chinese patient population with previously treated advanced NSCLC: CheckMate 078 randomized phase III clinical trial. J Thorac Oncol. 2019; 14(5): 867-875.
25. Schmid P, Adams S, Rugo H S, et al. Atezolizumab and nab-paclitaxel in advanced triple-negative breast cancer. N Engl J Med. 2018; 379(22): 2108-2121.
26. Finn R S, Qin S, Ikeda M, et al. Atezolizumab plus bevacizumab in unresectable hepatocellular carcinoma. N Engl J Med. 2020; 382(20): 1894-1905.
27. Rini B I, Powles T, Atkins M B, et al. Atezolizumab plus bevacizumab versus sunitinib in patients with previously untreated metastatic renal cell carcinoma (IMmotion151): a multicentre, open-label, phase 3, randomised controlled trial. Lancet. 2019; 393(10189): 2404-2415.
28. Rini B I, Plimack E R, Stus V, et al. Pembrolizumab plus axitinib versus sunitinib for advanced renal-cell carcinoma. N Engl J Med. 2019; 380(12): 1116-1127.
29. Zimmer L, Livingstone E, Hassel J C, et al. Adjuvant nivolumab plus ipilimumab or nivolumab monotherapy versus placebo in patients with resected stage IV melanoma with no evidence of disease (IMMUNED): a randomised, double-blind, placebo-controlled, phase 2 trial. Lancet. 2020; 395(10236): 1558-1568.
30. Gutzmer R, Stroyakovskiy D, Gogas H, et al. Atezolizumab, vemurafenib, and cobimetinib as first-line treatment for unresectable advanced BRAFV600 mutation-positive melanoma (IMspire150): primary analysis of the randomised, double-blind, placebo-controlled, phase 3 trial. Lancet. 2020; 395(10240): 1835-1844.
31. Galsky M D, Arija J Á A, Bamias A, et al. Atezolizumab with or without chemotherapy in metastatic urothelial cancer (IMvigor130): a multicentre, randomised, placebo-controlled phase 3 trial. Lancet. 2020; 395(10236): 1547-1557.
32. Govindan R, Szczesna A, Ahn M J, et al. Phase III trial of ipilimumab combined with paclitaxel and carboplatin in advanced squamous non–small-cell lung cancer. J Clin Oncol. 2017; 35(30): 3449-3457.
33. Lynch T J, Bondarenko I, Luft A, et al. Ipilimumab in combination with paclitaxel and carboplatin as first-line treatment in stage IIIB/IV non–small-cell lung cancer: results from a randomized, double-blind, multicenter phase II study. J Clin Oncol. 2012; 30(17): 2046-2054.
34. Robert C, Thomas L, Bondarenko I, et al. Ipilimumab plus dacarbazine for previously untreated metastatic melanoma. N Engl J Med. 2011; 364(26): 2517-2526.
35. Langer C J, Gadgeel S M, Borghaei H, et al. Carboplatin and pemetrexed with or without pembrolizumab for advanced, non-squamous non-small-cell lung cancer: a randomised, phase 2 cohort of the open-label KEYNOTE-021 study. Lancet Oncol. 2016; 17(11): 1497-1508.
36. Burtness B, Harrington K J, Greil R, et al. Pembrolizumab alone or with chemotherapy versus cetuximab with chemotherapy for recurrent or metastatic squamous cell carcinoma of the head and neck (KEYNOTE-048): a randomised, open-label, phase 3 study. Lancet. 2019; 394(10212): 1915-1928.
37. Mateos M V, Blacklock H, Schjesvold F, et al. Pembrolizumab plus pomalidomide and dexamethasone for patients with relapsed or refractory multiple myeloma (KEYNOTE-183): a randomised, open-label, phase 3 trial. Lancet Haematol. 2019; 6(9): e459-e469.
38. Usmani S Z, Schjesvold F, Oriol A, et al. Pembrolizumab plus lenalidomide and dexamethasone for patients with treatment-naive multiple myeloma (KEYNOTE-185): a randomised, open-label, phase 3 trial. Lancet Haematol. 2019; 6(9): e448-e458.
39. Long G V, Dummer R, Hamid O, et al. Epacadostat plus pembrolizumab versus placebo plus pembrolizumab in patients with unresectable or metastatic melanoma (ECHO-301/KEYNOTE-252): a phase 3, randomised, double-blind study. Lancet Oncol. 2019; 20(8): 1083-1097.
40. Schmid P, Cortes J, Pusztai L, et al. Pembrolizumab for early triple-negative breast cancer. N Engl J Med. 2020; 382(9): 810-821.
41. Maio M, Grob J J, Aamdal S, et al. Five-year survival rates for treatment-naive patients with advanced melanoma who received ipilimumab plus dacarbazine in a phase III trial. J Clin Oncol. 2015; 33(10): 1191.
42. Reck M, Luft A, Szczesna A, et al. Phase III randomized trial of ipilimumab plus etoposide and platinum versus placebo plus etoposide and platinum in extensive-stage small-cell lung cancer. J Clin Oncol. 2016; 34(31): 3740-3748.
43. Hodi F S, Lee S, McDermott D F, et al. Ipilimumab plus sargramostim vs ipilimumab alone for treatment of metastatic melanoma: a randomized clinical trial[J]. Jama, 2014, 312(17): 1744-1753.
44. Hellmann M D, Paz-Ares L, Bernabe Caro R, et al. Nivolumab plus ipilimumab in advanced non–small-cell lung cancer. N Engl J Med. 2019; 381(21): 2020-2031.
45. Fehrenbacher L, Spira A, Ballinger M, et al. Atezolizumab versus docetaxel for patients with previously treated non-small-cell lung cancer (POPLAR): a multicentre, open-label, phase 2 randomised controlled trial. Lancet. 2016; 387(10030): 1837-1846.
46. Powles T, Durán I, Van Der Heijden M S, et al. Atezolizumab versus chemotherapy in patients with platinum-treated locally advanced or metastatic urothelial carcinoma (IMvigor211): a multicentre, open-label, phase 3 randomised controlled trial. Lancet. 2018; 391(10122): 748-757.
47. Rittmeyer A, Barlesi F, Waterkamp D, et al. Atezolizumab versus docetaxel in patients with previously treated non-small-cell lung cancer (OAK): a phase 3, open-label, multicentre randomised controlled trial. Lancet. 2017; 389(10066): 255-265.
48. Weber J S, D'Angelo S P, Minor D, et al. Nivolumab versus chemotherapy in patients with advanced melanoma who progressed after anti-CTLA-4 treatment (CheckMate 037): a randomised, controlled, open-label, phase 3 trial. Lancet Oncol. 2015; 16(4): 375-384.
49. Kato K, Cho B C, Takahashi M, et al. Nivolumab versus chemotherapy in patients with advanced oesophageal squamous cell carcinoma refractory or intolerant to previous chemotherapy (ATTRACTION-3): a multicentre, randomised, open-label, phase 3 trial. Lancet Oncol.2019; 20(11): 1506-1517.
50. Ribas A, Puzanov I, Dummer R, et al. Pembrolizumab versus investigator-choice chemotherapy for ipilimumab-refractory melanoma (KEYNOTE-002): a randomised, controlled, phase 2 trial. Lancet Oncol. 2015; 16(8): 908-918.
51. Shitara K, Özgüroğlu M, Bang Y J, et al. Pembrolizumab versus paclitaxel for previously treated, advanced gastric or gastro-oesophageal junction cancer (KEYNOTE-061): a randomised, open-label, controlled, phase 3 trial. Lancet. 2018; 392(10142): 123-133.
52. Antonia S J, López-Martin J A, Bendell J, et al. Nivolumab alone and nivolumab plus ipilimumab in recurrent small-cell lung cancer (CheckMate 032): a multicentre, open-label, phase 1/2 trial. Lancet Oncol. 2016; 17(7): 883-895.
53. Hodi F S, Chiarion-Sileni V, Gonzalez R, et al. Nivolumab plus ipilimumab or nivolumab alone versus ipilimumab alone in advanced melanoma (CheckMate 067): 4-year outcomes of a multicentre, randomised, phase 3 trial. Lancet Oncol. 2018; 19(11): 1480-1492.
54. Wolchok J D, Chiarion-Sileni V, Gonzalez R, et al. Overall survival with combined nivolumab and ipilimumab in advanced melanoma. N Engl J Med. 2017; 377(14): 1345-1356.
55. Larkin J, Chiarion-Sileni V, Gonzalez R, et al. Five-year survival with combined nivolumab and ipilimumab in advanced melanoma. N Engl J Med. 2019; 381(16): 1535-1546.
56. Hodi F S, Chesney J, Pavlick A C, et al. Combined nivolumab and ipilimumab versus ipilimumab alone in patients with advanced melanoma: 2-year overall survival outcomes in a multicentre, randomised, controlled, phase 2 trial. Lancet Oncol. 2016; 17(11): 1558-1568.
57. Motzer R J, Tannir N M, McDermott D F, et al. Nivolumab plus ipilimumab versus sunitinib in advanced renal-cell carcinoma. N Engl J Med.2018;378(14):1277-1290
58. Motzer R J, Rini B I, McDermott D F, et al. Nivolumab plus ipilimumab versus sunitinib in first-line treatment for advanced renal cell carcinoma: extended follow-up of efficacy and safety results from a randomised, controlled, phase 3 trial. Lancet Oncol. 2019; 20(10): 1370-1385.
59. Scherpereel A, Mazieres J, Greillier L, et al. Nivolumab or nivolumab plus ipilimumab in patients with relapsed malignant pleural mesothelioma (IFCT-1501 MAPS2): a multicentre, open-label, randomised, non-comparative, phase 2 trial. Lancet Oncol. 2019; 20(2): 239-253.
60. Bang Y J, Cho J Y, Kim Y H, et al. Efficacy of sequential ipilimumab monotherapy versus best supportive care for unresectable locally advanced/metastatic gastric or gastroesophageal junction cancer. Clin Cancer Res. 2017; 23(19): 5671-5678.
61. Kwon E D, Drake C G, Scher H I, et al. Ipilimumab versus placebo after radiotherapy in patients with metastatic castration-resistant prostate cancer that had progressed after docetaxel chemotherapy (CA184-043): a multicentre, randomised, double-blind, phase 3 trial. Lancet Oncol. 2014; 15(7): 700-712.
62. Gandhi L, Rodríguez-Abreu D, Gadgeel S, et al. Pembrolizumab plus chemotherapy in metastatic non–small-cell lung cancer. N Engl J Med. 2018; 378(22): 2078-2092.
63. Paz-Ares L, Luft A, Vicente D, et al. Pembrolizumab plus chemotherapy for squamous non–small-cell lung cancer. N Engl J Med. 2018; 379(21): 2040-2051.
64. West H, McCleod M, Hussein M, et al. Atezolizumab in combination with carboplatin plus nab-paclitaxel chemotherapy compared with chemotherapy alone as first-line treatment for metastatic non-squamous non-small-cell lung cancer (IMpower130): a multicentre, randomised, open-label, phase 3 trial. Lancet Oncol. 2019; 20(7): 924-937.
65. Socinski M A, Jotte R M, Cappuzzo F, et al. Atezolizumab for first-line treatment of metastatic nonsquamous NSCLC. N Engl J Med. 2018; 378(24): 2288-2301.
66. Horn L, Mansfield A S, Szczęsna A, et al. First-line atezolizumab plus chemotherapy in extensive-stage small-cell lung cancer. N Engl J Med. 2018; 379(23): 2220-2229.
67. Paz-Ares L, Dvorkin M, Chen Y, et al. Durvalumab plus platinum–etoposide versus platinum–etoposide in first-line treatment of extensive-stage small-cell lung cancer (CASPIAN): a randomised, controlled, open-label, phase 3 trial. Lancet. 2019; 394(10212): 1929-1939.
